# Transition Metal‐Involved Photon Upconversion

**DOI:** 10.1002/advs.201600302

**Published:** 2016-11-29

**Authors:** Shi Ye, En‐Hai Song, Qin‐Yuan Zhang

**Affiliations:** ^1^ State Key Lab of Luminescent Materials and DevicesGuangdong Provincial Key Laboratory of Fiber Laser Materials and Applied TechniquesSouth China University of TechnologyGuangzhou510641China

**Keywords:** lanthanide ions, multifunctional materials, transition metal ions, tunable, upconversion

## Abstract

Upconversion (UC) luminescence of lanthanide ions (Ln^3+^) has been extensively investigated for several decades and is a constant research hotspot owing to its fundamental significance and widespread applications. In contrast to the multiple and fixed UC emissions of Ln^3+^, transition metal (TM) ions, e.g., Mn^2+^, usually possess a single broadband emission due to its 3*d* ^5^ electronic configuration. Wavelength‐tuneable single UC emission can be achieved in some TM ion‐activated systems ascribed to the susceptibility of *d* electrons to the chemical environment, which is appealing in molecular sensing and lighting. Moreover, the UC emissions of Ln^3+^ can be modulated by TM ions (specifically *d*‐block element ions with unfilled *d* orbitals), which benefits from the specific metastable energy levels of Ln^3+^ owing to the well‐shielded 4*f* electrons and tuneable energy levels of the TM ions. The electric versatility of *d* ^0^ ion‐containing hosts (*d* ^0^ normally viewed as charged anion groups, such as MoO_6_^6‐^ and TiO_4_^4‐^) may also have a strong influence on the electric dipole transition of Ln^3+^, resulting in multifunctional properties of modulated UC emission and electrical behaviour, such as ferroelectricity and oxide‐ion conductivity. This review focuses on recent advances in the room temperature (RT) UC of TM ions, the UC of Ln^3+^ tuned by TM or *d* ^0^ ions, and the UC of *d* ^0^ ion‐centred groups, as well as their potential applications in bioimaging, solar cells and multifunctional devices.

## 1 Introduction

**Figure 1:**
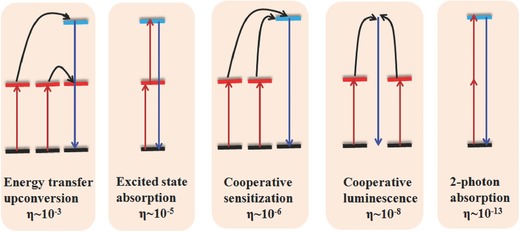
Some typical common UC processes and their relative efficiency η. Reproduced with permission.^1^ Copyright 2004, American Chemical Society.

Luminescence research has been performed for centuries, and it has increased the worldwide availability of artificial lighting and displays. Photon UC, known as anti‐Stokes emission, is a nonlinear optical phenomenon in which the sequential absorption of two or more low‐energy photons leads to high‐energy photon emission.^1^ The process appears magical, but Auzel proposed the occurrence of energy transfer to activators (luminescent centres) that are already in the excited state.^1^ This transfer is well established for activators in the ground state and can explain why *n* photons may be summed in the UC process. Some typical UC processes, such as energy transfer upconversion (ETU), excited state absorption (ESA) after ground state absorption (GSA), cooperative sensitisation, cooperative luminescence, two‐photon absorption, and the magnitude of their relative efficiency (all for the case of Ln^3+^), are schematically illustrated in **Figure** **1**.^1^ The ETU process can be described as two sensitisers sequentially transferring energy to a third ion with ladder‐like excited energy levels, resulting in the accumulation of energy and its release as high‐energy photons. The GSA/ESA process involves an activator absorbing one photon to excite the ground state to the first excited state, then absorbing another photon to reach the second excited state followed by UC emission. For cooperative sensitisation, two sensitisers simultaneously transfer their absorbed energy to the activator without an intermediate metastable state matched with a single pumping photon. Additionally, the activator can simultaneously absorb two photons in a two‐photon absorption process. In cooperative luminescence, two ions (such as Yb^3+^) pile up their absorbed photons to a virtual emitting level with subsequent luminescence. The ETU process has the highest efficiency among the four processes owing to the matching of the intermediate energy level of the activator and the excited state of the sensitiser for resonant energy transfer. Several UC processes may occur simultaneously in one system, the UC efficiency should be given along with the pumping power density data because it depends on the excitation intensity.

With the advent of powerful, inexpensive and readily available infrared (IR) laser diodes, there has been renewed interest in UC materials, despite the relatively low UC efficiency. The early discovered and well‐studied UC phenomena of Ln^3+^ have become conceptually appealing in many areas, including IR‐pumped bioapplications,^1^, ^2^, ^3^ displays,^4^ lasers^1^ and solar cells.^5^, ^6^, ^7^ In particular, IR‐pumped UC nanomaterials enable pumped light to penetrate tissue to a certain depth for imaging or probing of biosystems with reduced background interference. It is also a hot topic for the UC material layer on the reverse of a single‐junction bifacial solar cell to convert the transmitted sub‐bandgap photons into high‐energy photons, which can be absorbed again by the solar cell with resulting enhancement of the photo‐current conversion efficiency. For UC systems of Ln^3+^, Yb^3+^ has been extensively adopted as a sensitiser for Ln^3+^ in the UC process because of the strong oscillator strength of the ^2^ *F*_7/2_→^2^ *F*_5/2_ transition,^1^, ^2^, ^3^, ^4^, ^5^, ^6^, ^7^ which corresponds well with the excitation of the commercial 980 nm InGaAs laser diode. This characteristic of Yb^3+^ is a considerable advantage for sensitising Ln^3+^ with specific and less‐affected abundant metastable energy levels, which results in outstanding optical properties. However, the intentional modulation of UC behaviour is attractive for researchers to achieve the desired goals of specific applications. From this point of view, the less tuneable behaviour of the UC process is a disadvantage for Ln^3+^ because the 4*f* electrons are less affected by the chemical environment owing to the shielding of the outer shell 5*d* and 6*s* electrons.

**Figure 2:**
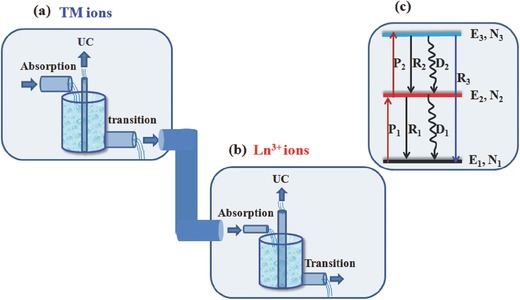
Optical characteristics sketch of a) TM ions and b) Ln^3+^ ions; c) simplified three‐energy‐level UC model with various transition processes involved.

In contrast, the optical behaviour of TM (specifically *d*‐block element ions with unfulfilled *d* orbitals) and *d* ^0^ ions (herein, *d* ^0^ ions are viewed as charged anion groups owing to the high‐valence states of *d*‐block elements, such as MoO_6_^6–^, VO_4_^3–^, and TiO_4_^4–^) can be extensively tuned because the outermost *d* electrons (there is a *d* electron for the excited state of *d* ^0^ ions) are strongly affected by their surrounding chemical environment. However, the UC of TM and *d* ^0^ ions is generally inefficient compared to that of Ln^3+^ and is only observed at cryogenic temperatures owing to the high non‐radiative transition probability of TM and *d* ^0^ ions in the UC process as the temperature increases.^1^, ^8^ **Figure** **2**a and 2b describe the transition characteristics of TM and Ln^3+^ ions with the assumed reservoirs. TM ions normally have broadband absorption but large non‐radiative relaxation rates and small UC rates, whereas Ln^3+^ has a relatively large UC rate and small non‐radiative transition rate but narrowband absorption. To elucidate how non‐radiative transition strongly influences the UC process, the simplified three‐energy‐level UC model (not considering the difference of ETU and ESA) in Figure 2c is utilised to determine the rate equation. Suppose that there are no other processes besides the ground‐state absorption *P*_1_, subsequent excited‐state absorption *P*_2_, depletion by radiative transitions *R*_1_, *R*_2_, and *R*_3_ and non‐radiative transitions *D*_1_ and *D*_2_. The population and depletion rates of each energy level *E* are proportional to their population density *N*. Ground‐state bleaching is assumed to be negligible, and ground state population *N*_1_ is constant. Then, the rate equation can be written as:^9^, ^10^, ^11^

(1) $$dN_{2}/dt\;=\;P_{1}N_{1}\;+\;R_{2}N_{3}\;+\;D_{2}N_{3}\;-\;R_{1}N_{2}\;-\;D_{1}N_{2}\;-\;P_{2}N_{2}$$

(2) $$dN_{3}/dt\;=\;P_{2}N_{2}\;-\;R_{2}N_{3}\;-\;D_{2}N_{3}\;-\;R_{3}N_{3}$$

For steady‐state excitation, d*N*_2_/dt = d*N*_3_/dt = 0, which yields:

(3) $$R_{3}N_{3}\;=\;P_{1}N_{1}\;-\;\left(R_{1}\;+\;D_{1}\right)N_{2}$$

Then, it can be concluded that UC luminescence (*R*_3_ *N*_3_) is primarily determined by the absorption efficiency (*P*_1_ *N*_1_) and the radiative (*R*_1_ *N*_2_) and non‐radiative (*D*_1_ *N*_2_) transition probabilities of the first metastable energy level *E*_2_, which indicates that a large absorption cross‐section (broad band) and spin‐ and parity‐allowed electric dipole transitions (*E*_1_→*E*_2_) are preferred for the first term *P*_1_ *N*_1_. The second term suggests that a metastable excited energy level with relatively long lifetime and weak electron‐phonon coupling is required. The deduction of these two terms appears to be conflicting for *E*_2_ but is reasonable if we take it not as a single energy level but as two levels from the respective sensitiser and activator for multicentre UC systems.^11^, ^12^, ^13^ From this point of view, TM ions are preferred as sensitisers.

**Figure 3:**
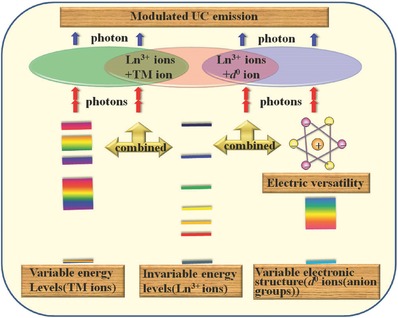
The schematically summarized approaches to modulate the UC behaviours of TM or *d* ^0^ ions (anion groups) and/or Ln^3+^ ions.

Despite the large multiphonon relaxation probabilities and luminescence quenching at RT for most TM ions, there are still some advances in the research on the RT UC of TM ions, especially those with tuneable single band NIR emission, such as Mn^2+^ and Cr^3+^, with relatively large gaps between the adjacent emitting level and ground state, which is attractive for bioimaging. When coupled with the multiphoton absorption of quantum dots (QDs)^14^, ^15^ or the metastable energy levels of Ln^3+^, it is possible to gain enhanced UC emission at RT or some other interesting UC phenomena. The UC process of Ln^3+^–TM ions benefits from the specific metastable energy levels of Ln^3+^, independent of the ligand field, owing to the well‐shielded 4*f* electrons and the tuneable energy levels of the TM ions resulting from the exposure of *d* electrons to the chemical coordination environment, a feature that is fascinating for many applications. The selection of different hosts and Ln^3+^/TM ions results in novel and unexpected UC properties, which leaves room for imaginative and creative applications.^1^, ^8^ Moreover, materials with TM or *d* ^0^‐centered anion groups have versatile and attractive functionalities in addition to their optical properties, including ferroelectric, magnetic and photocatalytic properties. By incorporating TM or *d* ^0^ ions into the crystal lattice with Ln^3+^, the UC behaviour of Ln^3+^ can be modulated via the electronic structure of the TM or the electric properties of the *d* ^0^‐contained hosts.^16^ Thus, UC materials with incorporated TM or *d* ^0^ ions (anion groups) exhibit fascinating multi‐functionality. The principles and approaches for achieving modulated UC behaviour of TM or *d* ^0^ ions (anion groups) and/or Ln^3+^ are schematically summarised in **Figure** **3**. There are several reviews on UC and its applications, but they almost exclusively focus on the UC of Ln^3+^.^2^, ^5^, ^7^, ^17^ Consequently, this review focuses on the recent advances in the RT UC behaviour of UC materials doped with TM or *d* ^0^ ions (anion groups) and Ln^3+^ and their potential applications, such as in solar cells,^5^, ^6^ optical temperature sensors,^18^ bioimaging through combined nuclear magnetic resonance and the UC method^19^ and photocatalysis,^20^ with an emphasis on the modulated UC behaviour.

## 2 Photon Upconversion of TM Ions

**Figure 4:**
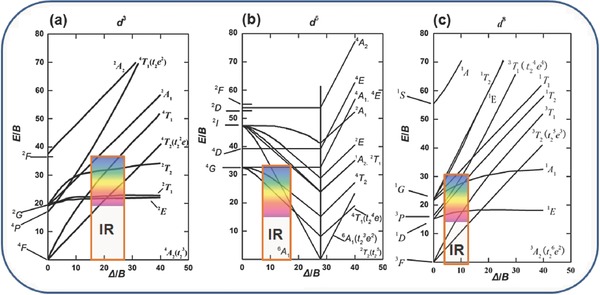
Tanabe‐Sugano diagram of commonly used *d* ^3^, *d* ^5^ and *d* ^8^ ions. The histograms roughly denote the gap energy between the excited state and ground state, in which the colour‐filled region and empty region represent that the energy levels locate at visible range and infrared (IR) range, respectively. Reproduced with permission.^25^ Copyright 1954, The Physical Society of Japan.

The tuneable single‐band feature is an advantage for some TM UC emissions and is strongly desirable for applications such as bioimaging. The UC behaviour of TM ions as either formula components or dopants, such as Mn^2+^, Cr^3+^, Re^4+^, V^3+^, Mo^3+^, Ni^2+^, and Ti^2+^, was extensively investigated at cryogenic temperatures by Auzel and Güdel et al.^1^, ^8^ However, their RT UC behaviour was rarely reported, except for that of Mn^2+^ and Cr^3+^. Generally, the electron‐phonon coupling effect for the *d* electrons of TM ions is larger than for the *f* electrons of Ln^3+^, resulting in a large non‐radiative transition possibility for TM ions. Therefore, the UC emission of TM ions could only be observed at cryogenic temperatures for most TM‐doped UC materials. For Mn^2+^ (3*d* ^5^) and Cr^3+^ (3*d* ^3^) ions, the emitting levels are normally the first excited states (^4^ *T*_1_ and ^4^ *T*_2_ or ^2^ *E*, respectively) with large gaps above the ground states (approximately 10,000–20,000 cm^–1^), as observed in **Figure** **4**a and 4b, which provides a greater possibility to prevent multiphonon relaxation, resulting in radiative emission at RT. The possibility of radiative transition between the upper excited state and lower excited state for TM ions is much smaller than that for Ln^3+^, which is also due to the electron‐phonon coupling. The cross‐relaxation results in greater quenching of the transition because there are abundant energy levels for TM ions, as in the case of Ni^2+^ (3*d* ^8^) and Re^4+^ (5*d* ^3^). The cross‐relaxation of ^1^ *T*_2_→^3^ *T*_2_+^3^ *A*_2_→^3^ *T*_1_ quenches the ^1^ *T*_2_→^3^ *A*_2_ green UC emission of Ni^2+^ in MgF_2_ when pumped by 752.5 nm laser,^21^ see Figure 4c. Re^4+^ was the first TM ion studied in UC material^22^ and the first TM ion to show RT UC phenomenon owing to the relaxation of the spin selection rules by its strong spin‐orbit coupling.^23^ The UC‐emitting state Γ_7_ (^2^ *T*_2g_) has a branching ratio of 1:2 for luminescence to ground state Γ_8_ (^4^ *A*_2g_) relative to the intermediate excited state Γ_8_ (^2^ *E*_g_). The cross‐relaxation process involving the Γ_8_ (^2^ *T*_1g_) state and Γ_8_ (^2^ *E*_g_) state would further reduce the UC luminescence efficiency.^24^

The spin‐forbidden transition characteristics of TM ions could be beneficial to the ESA UC process owing to the relatively long lifetime of the first excited state, for instance, the ^3^ *T*_1_→^1^ *T*_2_ transition of Ti^2+^ (*d* ^2^) in MgCl_2_.^26^ The spin‐allowed transition characteristics of TM ions benefit the broadband sensitisation of UC ions, such as the ^3^ *T*_1g_→^3^ *T*_2g_ of V^3+^ (*d* ^2^) and ^2^ *A*_1_→^2^ *B*_2_ of Cr^5+^ (*d* ^1^) in V^3+^–Re^4+^,^27^ V^3+^–Mo^3+^,^28^ V^3+^–Er^3+^,^29^ V^3+^–Pr^3+^ 30 and Cr^5+^–Er^3+^ 31 systems.

Sensitisation of the UC emission of TM ions with another TM or Ln^3+^ in codoped systems requires the emitted TM ion to absorb the sensitising energy from another TM or Ln^3+^, but the latter cannot absorb the energy emitted by the former. This requirement for energy level matching rules out many TM–TM and Ln–TM couples because they have abundant energy levels in the full range of the visible and infrared regions. Furthermore, there are forward and backward energy transfer processes in these codoped systems. Yb^3+^, with only one simple excited state at ≈10,000 cm^–1^, is an unique sensitiser for TM ions, especially for near‐infrared (NIR) to visible (VIS) UC emission.

### 2.1 Visible Upconversion of Mn^2+^

**Figure 5:**
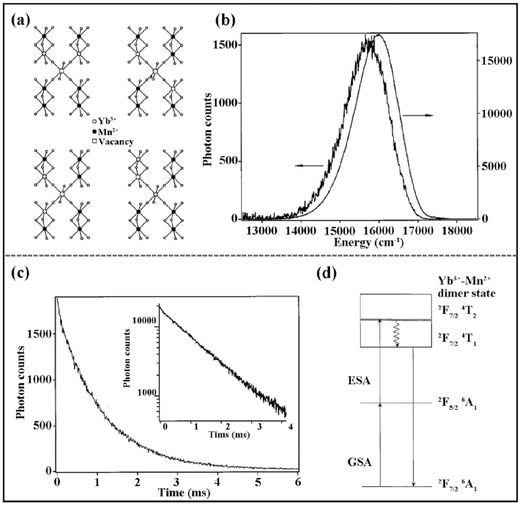
a) Possible arrangements of Yb^3+^ ions in pairs with charge compensation vancancies; b) UC (thick line) and Stokes (thin line) luminescence spectra of RbMnCl_3_:Yb^3+^ under CW excitation at 10686 and 19436 cm^–1^, respectively, at 10 K; c) Temporal behavior of Mn^2+^ UC emission after excitation of a pulsed laser light ≈10686 cm^–1^ at 15 K, inset shows the same data in a semilogarithmic scale; d) The proposed UC luminescence process. Reproduced with permission.^32^ Copyright 2001, The American Physical Society.

The UC of Mn^2+^ sensitised by Yb^3+^ was initially unexpected because Yb^3+^ has no *f* excited state above ^7^ *F*_5/2_ (≈10,000 cm^–1^), whereas Mn^2+^ has no *d* excited state below ^4^ *T*_1_ (≈17,000 cm^–1^).^8^ Therefore, the UC of Mn^2+^–Yb^3+^ is achieved via a cooperative sensitisation mechanism or exchange‐coupled Mn^2+^–Yb^3+^ dimer model. Bromide and chloride hosts with highly concentrated Mn^2+^ were used to guarantee the neighbouring Mn^2+^–Yb^3+^ in the first reported UC cases, ensuring the possibility of a superexchange interaction. There is a tendency for trivalent impurities to cluster in pairs owing to the charge compensation requirements in the linear‐chain lattice when trivalent Ln^3+^ substitutes for divalent cations.^32^ For instance, in the RbMnCl_3_:Yb^3+^ system, three neighbouring Mn^2+^ ions are replaced by two Yb^3+^ ions with one divalent vacancy as charge compensation,^32^ as schematically illustrated in **Figure** **5**a. Thus, the UC of Mn^2+^ can be observed at cryogenic temperature, see Figure 5b. When directly exciting Mn^2+^, the Stokes luminescence of Mn^2+^ shows an apparent redshift compared to the UC emission peak when exciting Yb^3+^ in Figure 5b because the former is contributed by the Mn^2+^ in the system, whereas the latter is contributed by the Mn^2+^ in the vicinity of Yb^3+^ by superexchange interaction. The temporal decay curve in Figure 5c without a rising stage suggests that the UC mechanism of Mn^2+^–Yb^3+^ in this RbMnCl_3_:Yb^3+^ system is GSA/ESA (Figure 5d). Laser light excites the Mn^2+^–Yb^3+^ pair from the |^2^ *F*_7/2_,^6^ *A*_1_> ground state into the |^2^ *F*_5/2_,^6^ *A*_1_> intermediate excited state in the GSA step. In the following ESA step, the pair is further promoted from the |^2^ *F*_5/2_,^6^ *A*_1_> state into the |^2^ *F*_7/2_,^4^ *T*_1_> emitting state and emits a visible photon. The detailed UC luminescence processes are as follows:

$${|}^{2}F_{7/2}{,}^{6}A_{1}\;>\;+h\nu \left(936\;\;\mathrm{nm}\right)\to {|}^{2}F_{5/2}{,}^{6}A_{1}\;>\;\left(\mathrm{GSA}\right)$$

$${|}^{2}F_{5/2}{,}^{6}A_{1}\;>\;+h\nu \left(936\;\;\mathrm{nm}\right)\to {|}^{2}F_{7/2}{,}^{4}T_{1}\;>\;\left(\mathrm{ESA}\right)$$

$${|}^{2}F_{7/2}{,}^{4}T_{1}\;>\;\to {|}^{2}F_{7/2}{,}^{6}A_{1}\;>\;+h\nu \left(\mathrm{VIS}\right)$$

The UC efficiency of the exchange‐coupled Mn^2+^–Yb^3+^ dimer is configuration geometry‐dependent.^33^ The ratio of the Mn^2+^ UC and the Yb^3+^ NIR was taken as a measure of the UC efficiency when excited by a 980 nm laser. **Table** **1** shows that the observed UC efficiency decreases by orders of magnitude from corner‐sharing bridging in Rb_2_MnCl_4_:Yb^3+^ to edge‐sharing in MnCl_2_:Yb^3+^/MnBr_2_:Yb^3+^ to face‐sharing in CsMnBr_3_:Yb^3+^. The coupling interaction may be weakened when the Mn^2+^–Yb^3+^ distance increases but is strengthened by the linear configuration.

**Table 1:** Comparison of (Mn^2+^/Yb^3+^) polyhedral bridging geometry, Mn^2+^–Yb^3+^ distance, Mn–L–Yb angle (L = halogen ions) and UC efficiency (ratio of the Mn^2+^ UC emission to the Yb^3+^ NIR emission was used when a 191 mW laser beam was focused on the sample by an *f* = 53 mm lens)^33^

Compound	Bridging geometry	Mn^2+^–Yb^3+^ distance[Å]	Mn–L–Yb angle[°]	η_UC_[%]	T[K]	Ref.
Rb_2_MnCl_4_:Yb^3+^	corner	5.05	180	28(site A)	35	34
Rb_2_MnCl_4_:Yb^3+^	corner	5.05	180	18(site B)	15	34
CsMnCl_3_:Yb^3+^	corner	5.2	177.2	8.5	75	35
RbMnCl_3_:Yb^3+^	corner	5.02	177.1	2	10	32
MnCl_2_:Yb^3+^	edge	3.71	92.8	4.1	12	33
MnBr_2_:Yb^3+^	edge	3.82	89.8	1.2	12	33
RbMnCl_3_:Yb^3+^	face	3.2	77.9	0.02	10	32
CsMnBr_3_:Yb^3+^	face	3.26	74.8	0.05	12	36
RbMnBr_3_:Yb^3+^	face	3.37	76.1	0.05	12	33

**Figure 6:**
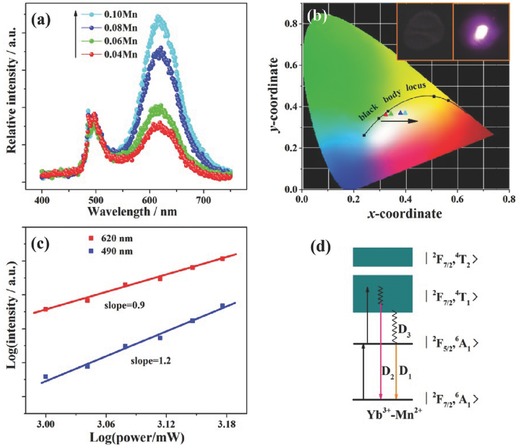
a) The UC luminescence of Gd_0.96_Mg_1–x_B_5_O_10_: *x*Mn^2+^, 0.04Yb^3+^ upon the excitation of a 976 nm laser diode; b) The CIE chromaticity coordinates for UC luminescence of Gd_0.96_Mg_1–x_B_5_O_10_: *x*Mn^2+^, 0.04Yb^3+^ samples. Red, green, blue and cyan stars denote the samples with *x* = 0.04, 0.06, 0.08 and 0.10, respectively. Insert shows the photos of the sample(*x* = 0.04) with and without 976 nm laser excitation; c) Power dependency of Gd_0.96_Mg_0.96_B_5_O_10_: 0.04Mn^2+^, 0.04Yb^3+^ luminescence intensities; d) The proposed UC luminescence mechanism. Reproduced with permission.^10^ Copyright 2011, The Royal Society of Chemistry.

**Figure 7:**
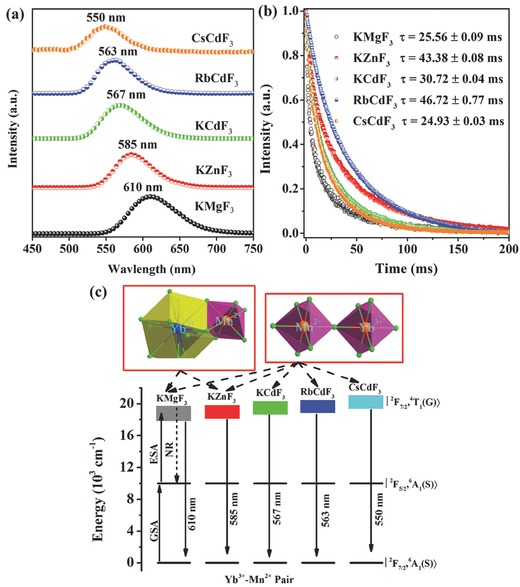
a) UC emission spectra and b) UC decay curves of ABF_3_:0.005Yb^3+^, 0.05Mn^2+^(A = K^+^, Rb^+^ and Cs^+^; B = Mg^2+^, Zn^2+^ and Cd^2+^) upon excitation of a 976‐nm Laser diode; c) The proposed UC luminescence mechanism. Reproduced with permission.^43^

The UC luminescence of Mn^2+^–Yb^3+^ in the above systems is only observed at cryogenic temperature. One possible reason may be the low‐lying red emitting state of Mn^2+^, which is above the ^7^F_5/2_ state of Yb^3+^, resulting in multiphonon relaxation between the two states. The higher‐lying emitting state of Mn^2+^ in the green region could be responsible for the RT UC phenomenon, which occurs in Zn_2_SiO_4_:Yb^3+^,Mn^2+^.^37^ Another possible reason to be considered is that the highly‐concentrated Mn^2+^ would significantly quench the luminescence in most of the cases. It was initially and intentionally designed for the systems with highly‐concentrated Mn^2+^ in the early reports of Table 1 to ensure the vicinity of Yb^3+^ and Mn^2+^ ions. Recently, RT green or red UC of Mn^2+^ has been observed in many other hosts with diluted Mn^2+^, such as LaMgAl_11_O_19_:Mn^2+^,Yb^3+^ aluminates,^38^ GdMgB_5_O_10_:Mn^2+^,Yb^3+^ borates,^10^ BaB_8_O_13_:Mn^2+^,Yb^3+^ borates,^39^ MgGa_2_O_4_:Mn^2+^,Yb^3+^ gallates,^40^ and KZnF_3_:Mn^2+^,Yb^3+^ perovskite fluorides.^41^ The emission of Mn^2+^ is strongly affected by the chemical coordination environment that accommodates the Mn^2+^ ion. Green emission of Mn^2+^ at ≈514 nm is observed when it replaces Mg^2+^ in LaMgAl_11_O_19_:Mn^2+^,Yb^3+^, which is tetrahedrally coordinated with oxygen ions.^38^ When Mn^2+^ substitutes for Mg^2+^ in the MgO_6_ octahedron of GdMgB_5_O_10_:Mn^2+^,Yb^3+^, it emits red light at ≈620 nm.^10^ Moreover, white‐light emission composed of two broadbands with peaks at 490 and 620 nm is achieved in GdMgB_5_O_10_:Mn^2+^,Yb^3+^, as observed in **Figure** **6**a and 6b, originating from the upconverted emissions of Yb^3+^–Yb^3+^ and Yb^3+^–Mn^2+^ dimers, respectively. The cooperative luminescence and sensitisation mechanisms are excluded for these two dimers according to the luminescence behaviours, and exchange interaction models are proposed for these two‐photon processes based on the crystal structure. The large deviations from two of the plot slopes (1.2 and 0.9) of the UC luminescence intensity as a function of pump power in Figure 6c are quantitatively interpreted by the large UC rate for both cases and an additional depletion pathway of relaxation from the upper excited state |^2^ *F*_7/2_,^4^ *T*_1_> to the intermediate state |^2^ *F*_5/2_,^6^ *A*_1_> for the Yb^3+^–Mn^2+^ dimer (Figure 6d) according to the exchange interaction model and rate equations.^9^, ^10^ The UC luminescence colour can be tuned by varying the amounts of Yb^3+^ and Mn^2+^, indicating that GdMgB_5_O_10_:Yb^3+^, Mn^2+^ is a potential candidate for lighting and displays. It was recently demonstrated that RT Mn^2+^ UC emission could be tuned from 550 to 610 nm in Yb^3+^/Mn^2+^ codoped fluoride perovskite homologous compounds ABF_3_ (A = K^+^, Rb^+^ and Cs^+^; B = Mg^2+^, Zn^2+^ and Cd^2+^), as shown in **Figure** **7**a, depending on the A^+^ and/or the B^2+^ cations.^42^, ^43^ The different bond length of (B^2+^/Mn^2+^)–F^–^ causes distinct crystal field strength on Mn^2+^, resulting in a different wavelength for Mn^2+^. These UC emissions have ultra‐long decay lifetimes (Figure 7b). Because Yb^3+^ may substitute both the A^+^ and the B^2+^ cations in KMgF_3_ and KZnF_3_, two exchange‐coupling geometry models for Yb^3+^–Mn^2+^ UC luminescence are proposed in Figure 7c. For the other hosts, Yb^3+^ could only substitutes B^2+^ cation according to the luminescence data, which was also found in previous work. In contrast to the well‐known multiple and fixed UC emissions from Ln^3+^ activated materials, the room (or high) temperature UC emission and ultra‐long UC decay lifetimes (≈25–45 ms) of the ABF_3_:Yb^3+^,Mn^2+^ perovskites suggest that they have potential applications in time‐resolved luminescence imaging, lighting and solid‐state lasers.

UC emission of Mn^2+^ could also be sensitised by the semiconductor QDs via multiphoton excitation. Multiphoton excitation, which is a process that occurs when multiple photons are simultaneously absorbed within QDs through virtual states, is a common phenomenon for semiconductor QDs. Normally, the emission of QDs in this multiphoton excitation process is intraband transition between the conduction band and the valence band. When doping with Mn^2+^, UC emission of Mn^2+^ can be observed. Take ZnS:Mn^2+^ for an example; strong UC luminescence of Mn^2+^ is observed in bulk and nanoparticle ZnS:Mn^2+^.^14^ Based on the experimental data of the quadratic power dependence of UC emission and the nearly identical luminescence profiles and lifetimes upon excitation at 385.5 and 767 nm, the authors concluded that the UC luminescence of ZnS:Mn^2+^ occurred through a two‐photon process, even though there is no energy level at the excitation wavelength of 767 nm for either ZnS or the Mn^2+^ ion. Further UC emission experiments of Eu^3+^‐codoped ZnS:Mn^2+^ provided evidence of the two‐photon absorption mechanism.^15^ The emission profiles for UC and Stokes luminescence when exposed to UC excitation wavelengths and half of the UC excitation wavelength (double the energy) are analogous in this system. However, a change of a few nanometres in the excitation wavelength results in a dramatic change in the UC emission intensity for Eu^3+^single‐doped ZnS, indicating that there is a mismatch between the Eu^3+^ energy levels and double the energy of the UC excitation wavelength. Most recently, three‐photon‐excited UC luminescence of ZnS:Mn^2+^ nanocrystals was proposed based on the slope (≈2.9) of the Mn^2+^ UC emission intensity as a function of the incident power.^44^ The large three‐photon cross‐section of ZnS QDs results in high spatial resolution for targeted cellular imaging, and the three‐photon process modulated by Mn^2+^ with visible red emission enables its application in high‐resolution in vivo deep‐tissue imaging without ultraviolet‐induced photo‐damage.

### 2.2 Near‐Infrared Upconversion of Mn^2+^

**Figure 8:**
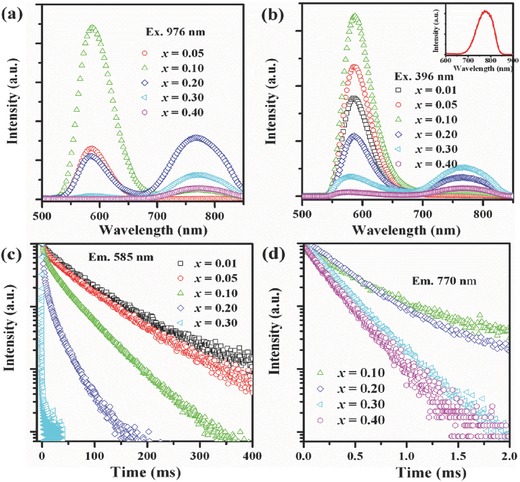
a) UC emission spectra of KZn_0.995–_ *_x_*Mn*_x_*Yb_0.005_F_3_ (*x* = 0.05–0.40) upon excitation of a 976 nm laser diode; b) Emission spectra of KZn_0.995–_ *_x_*Mn*_x_*Yb_0.005_F_3_ (*x* = 0.05–0.40) upon excitation of 396 nm, inset shows the emission spectrum of KMnF_3_; Luminescence decay curves of c) 585 nm and d) 770 nm emission for KZn_1–_ *_x_*Mn*_x_*F_3_(*x* = 0.01–0.40) upon 396 nm excitation. Reproduced with permission.^45^

Single NIR UC emission is highly preferred in bioimaging applications because it can penetrate deeper into tissue with less noise. Traditionally, Mn^2+^ has been considered as a VIS emitting ion. Most recently, abnormal NIR emission of Mn^2+^ at RT was reported in addition to the normal VIS emission.^42^, ^45^ For KZn_0.995–x_Mn_x_Yb_0.005_F_3_ systems at a low Mn^2+^ concentration (*x* = 0.05), the emission spectrum consists of a single VIS UC emission centred at 585 nm, as shown in **Figure** **8**a, corresponding to the |^2^ *F*_7/2_,^4^ *T*_1_>→ |^2^ *F*_7/2_,^6^ *A*_1_> transition of the Yb^3+^–Mn^2+^ dimers. In contrast, an additional anomalous NIR emission band centred at 770 nm emerges in the luminescence spectra in Figure 8a and 8b when the doping concentration of Mn^2+^ is sufficiently high (*x* = 0.10–0.40).^45^ Figure 8c and 8d demonstrate that the two emission peaks exhibit distinct decay behaviour, which suggests that the peaks originate from different emission centres. Usually, Mn^2+^ in a solid matrix exhibits only visible photoluminescence owing to the relatively large energy gap (>17,000 cm^–1^) between the first excited level and the ground state of the 3*d* ^5^ electronic configuration. Normally, when the electrons of Mn^2+^ are excited to energy levels above the ^4^ *T*_1_ (^4^ *G*) emitting state, relaxation occurs non‐radiatively from the higher states until the emitting state is reached. This transition strongly depends on the crystal field of the ligands, which is most visible in the characteristic green luminescence typically associated with tetrahedral‐coordinated Mn^2+^ (i.e., weaker ligand field) and the orange or red luminescence from octahedral Mn^2+^ (i.e., stronger ligand field).^46^ NIR emission has recently been observed in association with Mn^2+^ doping at elevated Mn^2+^ concentrations, as observed in Figure 8, which is not currently understood because only one large energy gap between the ground state and the first excited state of Mn^2+^ is known to cause luminescence. There is only a single, well‐defined lattice site that Mn^2+^ can possibly occupy in this simple cubic perovskite compound. One possible explanation for this concentration‐dependent behaviour is the occurrence of Mn^2+^ ion aggregation. The delocalisation and interaction of the 3*d* electrons of Mn^2+^ may result in novel luminescent centres with unusual luminescence behaviour. The novel NIR UC emission has been experimentally and theoretically investigated in Yb^3+^/Mn^2+^ codoped KMgF_3_ perovskites.

**Figure 9:**
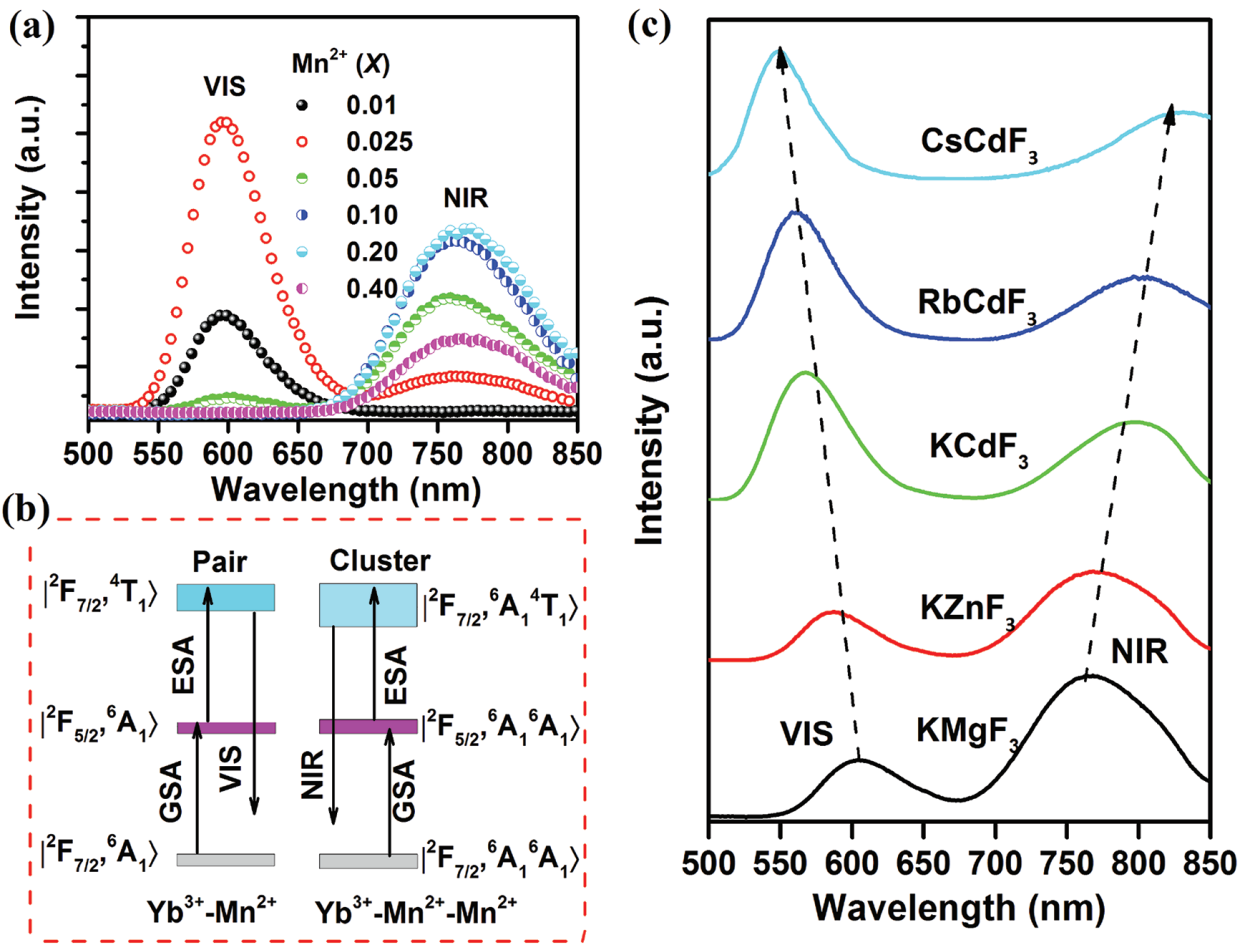
a) RT UC emission spectra of KMg_0.995–x_F_3_:0.005Yb^3+^,xMn^2+^ excited by a 976 nm laser diode at a power density of 10 W cm^–2^; b) The proposed VIS and NIR UC emission mechanism in KMgF_3_:Yb^3+^,Mn^2+^; c) UC emission spectra of ABF_3_:20%Mn^2+^,0.5%Yb^3+^ (A = K, Rb, Cs; B = Mg, Zn, Cd). Reproduced with permission.^42^

The Mn^2+^ concentration‐dependent photoluminescence phenomena of KMgF_3_:Yb^3+^,Mn^2+^ nanocrystals^42^ are illustrated in **Figure** **9**a, which is analogous to Figure 8. First‐principle calculations of the lattice geometry of possible substitution models revealed that the model with the shortest Mn^2+^–Mn^2+^ distance and antiferromagnetic (AFM) interaction has the lowest formation energy, which suggests that Mn^2+^ aggregation may occur.^42^ This is in agreement with the previously reported experimental observation of AFM in KMnF_3_ and KMgF_3_:Mn^2+^. The extended X‐ray absorption fine structure (EXAFS) provides experimental evidence for Mn^2+^ ion aggregation in KMgF_3_:Mn^2+^, which is in good agreement with the luminescence behaviour of KMgF_3_:Mn^2+^ with increasing Mn^2+^ content. The aggregation‐induced, geometry‐dependent coupling of Mn^2+^ is indicated by the temperature‐dependent emission spectra of KMnF_3_, NaMnF_3_ and CsMnF_3_, which provide different Mn^2+^–Mn^2+^ linkage geometry because of the different radii of the alkali ions.^42^ Therefore, UC models for VIS and NIR emission in this system are proposed in Figure 9b based on the Yb^3+^–Mn^2+^ dimer and Yb^3+^–Mn^2+^–Mn^2+^ trimer images, respectively, with the GSA/ESA mechanism. Furthermore, the ligand‐field‐dependent luminescence behaviour of these emission centres was also investigated.^42^ Figure 9c presents the UC emission spectra of ABF_3_:20%Mn^2+^,0.5%Yb^3+^ upon excitation with a 976 nm laser diode. The emission spectra comprise both VIS and NIR emission. The VIS UC emission occurs at 605, 585, 567, 563 and 550 nm in the Yb^3+^/Mn^2+^ codoped KMgF_3_, KZnF_3_, KCdF_3_, RbCdF_3_ and CsCdF_3_, respectively. In addition, the corresponding NIR UC emission is centred at 765, 770, 795, 805 and 830 nm. These results demonstrate that the UC emission centres of Yb^3+^–Mn^2+^ dimers (pairs) and Yb^3+^–Mn^2+^–Mn^2+^ trimers have an intrinsic formation tendency in these Yb^3+^/Mn^2+^‐doped perovskite structures. Both the VIS and NIR emissions could be finely tuned. The VIS emission monotonically blueshifts with an increase in the lattice constant owing to the decrease of crystal field strength. However, the NIR emission gradually shifts in the opposite direction with increasing lattice constant. The NIR/VIS emission ratio decreases monotonically with increasing lattice constant, indicating that the Mn^2+^–Mn^2+^ dimers are preferentially formed in the host lattice with shorter Mn^2+^–Mn^2+^ distance. The NIR emission band may result from the ^6^ *A*_1g_(*S*)^4^ *T*_1g_(*G*)→^6^ *A*_1g_(*S*)^6^ *A*_1g_(*S*) transition of the coupled Mn^2+^–Mn^2+^ dimers, which is a spin‐allowed transition with a decay lifetime shorter than 0.50 ms. NIR emissions have also been found in other Mn^2+^–Yb^3+^ doping systems, such as MgGa_2_O_4_: Mn^2+^,Yb^3+^ gallates.^40^ The result is fascinating because the observed NIR UC emission is located in the “optical window” of living cells and tissues, which provides an opportunity to achieve high‐resolution and deep penetration in biological imaging. The present results not only provide a useful and effective approach for obtaining pure NIR UC emission but also new perspective on the development of advanced photonic devices and technologies.

### 2.3 Upconversion of Cr^3+^

**Figure 10:**
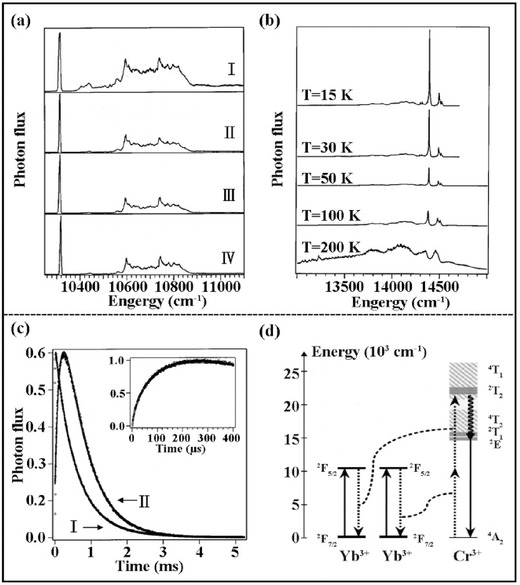
a) Excitation spectra (I–III) of Y_3_Ga_5_O_12_(YGG):2%Cr^3+^,1%Yb^3+^ at 15 K monitoring the NIR Yb^3+^ (at 9770 cm^–1^),the red Cr^3+^ (at 14388 cm^–1^) and the green Yb^3+^–Yb^3+^ luminescence (at 20628 cm^–1^), respectively. IV shows the square of spectrum in I; b) Temperature dependence of the UC luminescence spectra of the sample upon 10314 cm^–1^ laser excitation; c) time evolution of the sample following 10 ns pulsed excitation with the excitation wavelength of 14641 cm^–1^(I) and 10314 cm^–1^(II) at 15 K. The inset shows the enlarge scale of rise‐up in (II); d) UC mechanism scheme: straight up, dashed, curly and straight down arrows represent excitation, nonradiative energy transfer, nonradiative multiphonon relaxation and luminescence steps, respectively. Reproduced with permission.^47^ Copyright 2002, The American Physical Society.

Cr^3+^ is another commonly used TM luminescent centre with efficient deep red or NIR emission that can be cooperatively sensitised by Yb^3+^ to achieve UC emission in YAlO_3_, Y_3_Al_5_O_12_ and Y_3_Ga_5_O_12_.^8^ Cr^3+^ has no excited states below 14,000 cm^–1^, whereas Yb^3+^ has none above 10,000 cm^–1^. However, the UC of Yb^3+^–Cr^3+^ was observed, as shown for Y_3_Ga_5_O_12_: Yb^3+^,Cr^3+^ in **Figure** **10**.^47^ The excitation spectra of Yb^3+^ NIR luminescence, Yb^3+^–Yb^3+^ green UC emission and Cr^3+^ red UC emission have similar profiles in Figure 10a, which is ascribed to the absorption of Yb^3+^: ^2^ *F*_7/2_→^2^ *F*_5/2_. The sharp UC emission ^2^ *E*→^4^ *A*_2_ of Cr^3+^ gradually changed to a broad UC emission of ^4^ *T*_2_→^4^ *A*_2_ of Cr^3+^ with increasing temperature, as shown in Figure 10b, because the ^4^ *T*_2_ state could be thermally populated at elevated temperature. The smaller gap between the ^4^ *T*_2_ state of Cr^3+^ and the ^2^ *F*_5/2_ state of Yb^3+^ (with a reference gap between the ^2^ *E* state of Cr^3+^ and the ^2^ *F*_5/2_ state of Yb^3+^) causes stronger temperature quenching in this system. The apparent increase in the early stage of the UC decay curve in Figure 10c suggests the occurrence of energy transfer. Because the Yb^3+^–Yb^3+^ dimer is not likely formed in this garnet structure, the excited energy of two Yb^3+^ ions could simultaneously transfer to the Cr^3+^ in their vicinity, as demonstrated in Figure 10d. This so‐called cooperative sensitisation mechanism requires the overlap of an excited state with twice the absorbed energy of Yb^3+^.

**Figure 11:**
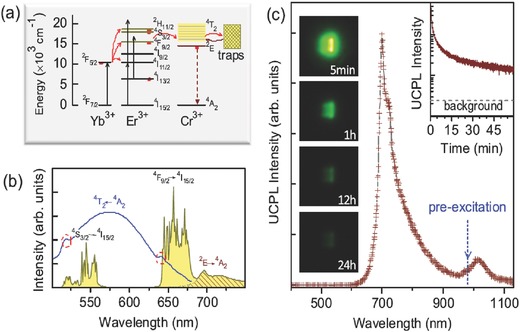
a) UCPL schematic diagram of Zn_3_Ga_2_GeO_8_:Yb^3+^,Er^3+^,Cr^3+^. The straight‐line arrows and curved line arrows represent optical transitions and energy (or electron) transfer processes, respectively. b) UC emission spectrum (curve with yellow shadow) upon excitation of a 980 nm laser and excitation spectrum of Cr^3+^ (blue solid‐line curve) by monitoring the 700 nm emission. The red dashed‐line circles indicate the positions of the characteristic excitation peaks of Er^3+^. The Cr^3+^ emission was filled by the diagonal shadow. c) UCPL emission spectrum of Zn_3_Ga_2_GeO_8_:Yb^3+^,Er^3+^,Cr^3+^ at 5 s after the stoppage of a 980 nm laser excitation. The upper right insert is the UCPL decay curve monitored at 700 nm emission. The left insert shows the NIR images (false color) taken at different delay times (5 min to 24 h) after ceasing the 980 nm laser excitation. For all the UCPL measurements, the samples were irradiated by a 980 nm laser for 10 min at a power of 600 mW. Reproduced with permission.^49^ Copyright 2014, American Physical Society.

However, RT UC emission of Cr^3+^ has rarely been reported. Recently, RT UC emission of Cr^3+^ has been achieved via energy transfer from Ln^3+^ ions when pumped by a 980 nm laser diode, such as La_3_Ga_5_GeO_14_:Yb^3+^,Er^3+^,Cr^3+^,^45^, ^48^ La_3_Ga_5_GeO_14_:Yb^3+^,Tm^3+^,Cr^3+^,^49^ and Zn_3_Ga_2_GeO_8_:Yb^3+^,Er^3+^,Cr^3+^.^49^ When introducing the new function of persistent luminescence in the UC emission of Cr^3+^, the novel phenomenon of upconverted persistent luminescence (UCPL) has been conceptually demonstrated by combining Ln^3+^ (with outstanding UC performance) with Cr^3+^ (with excellent persistent luminescence) in Zn_3_Ga_2_GeO_8_:Yb^3+^,Er^3+^,Cr^3+^.^49^ The unique NIR UCPL emission is ascribed to the ^2^ *E*→^4^ *A*_2_ transition of Cr^3+^, as shown in **Figure** **11**. The directly observed phenomenon is the afterglow emission after stoppage of a 980 nm laser for the samples preheated for 20 min at 400 °C to completely empty the electron traps. The UC emission of Yb^3+^–Er^3+^ is firstly absorbed by Cr^3+^, as proved by the overlap between the excitation spectrum of Cr^3+^ and the emission spectrum of Er^3+^ in Figure 11b. Then, the absorbed energy transfers to the trap centres and is finally released in the form of Cr^3+^ NIR emission by thermal activation. The VIS UC emission of Er^3+^ induced persistent luminescence of Cr^3+^, as shown by the similar persistent luminescence induced by a red LED light.^49^ The emission peak at ≈1000 nm in Figure 11c is due to the energy transfer from Cr^3+^ to Yb^3+^. More evidence for the UCPL phenomenon is that different duration times of the 980 nm laser cause distinct thermoluminescence (TL) peaks in this Zn_3_Ga_2_GeO_8_:Yb^3+^,Er^3+^,Cr^3+^ system, and they are also different from the TL behaviour excited by UV or visible light. It suggests that 980 nm laser illumination not only produces upconverted excitation to fill the traps but also influences the electron distribution in the traps. While the shallow traps are capturing electrons from the excited Cr^3+^, the 980 nm excitation is also promoting the trapped electrons to the delocalised conduction band, resulting in depopulation of electrons in the shallow traps.^49^ Conceptually, this NIR UCPL offers the potential of excitation‐free and noise‐free deep tissue in vivo imaging for bioapplications.

**Figure 12:**
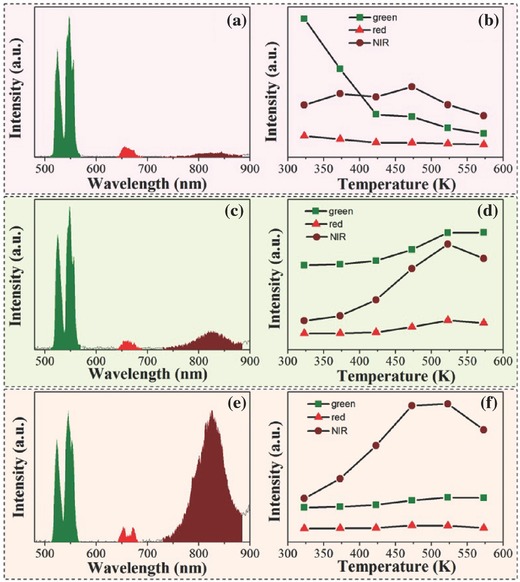
UC Luminescence spectra and corresponding integrated intensity of the three emission peaks of LGG (short for La_3_Ga_5_GeO_14_): xCr^3+^, 0.12Yb^3+^, 0.06Er^3+^ (for a and b, x = 0.02; for c and d, *x* = 0.06; for e and f, *x* = 0.15). Reproduced with permission.^48^ Copyright 2015, The Royal Society of Chemistry.

However, UCPL was not observed in La_3_Ga_5_GeO_14_:Cr^3+^,Yb^3+^,Er^3+^, even though there was NIR persistent luminescence of Cr^3+^ and energy transfer from the UC of Er^3+^ to Cr^3+^.^48^ Therefore, the temperature‐dependent UC luminescence of Er^3+^ and Cr^3+^ in this system was investigated in detail, and the results are presented in **Figure** **12**. The NIR UC luminescence at ≈830 nm is ascribed to the ^4^ *T*_2_→^4^ *A*_2_ transition of Cr^3+^. The NIR emission intensities of all samples show an initial increase followed by a decline with increasing temperature, whereas that of the UC green emission of Er^3+^ behaves distinctly for samples with different concentrations of Cr^3+^. For the sample with low concentration (*x* = 0.02) of Cr^3+^ (Figure 12b), it decreases as the temperature rises, whereas for the sample with a higher concentration (*x* = 0.06) of Cr^3+^ (Figure 12d), there is a distinct initial increase followed by a decrease as the temperature rises. For the sample with a high concentration (*x* = 0.15) of Cr^3+^ (Figure 12f), it varies little with temperature. Additionally, the red emission intensity does not vary dramatically with temperature. Normally, thermal quenching behaviour is expected as the temperature rises because the probability of multi‐phonon relaxation and energy transfer to quench the emitting levels is enhanced. However, anti‐thermal‐quenching behaviour was observed in this system, as shown in Figure 12. A possible reason for this phenomenon is that there may be TL in this system. The sample shows a TL peak, but after heating, there is a horizontal line in the temperature range from 313 to 473 K. When the sample is excited by a 976 nm laser for 5 min in a black box, the TL spectrum remains as a horizontal line. The TL peak reappears with sample exposure to simulated sunlight for 5 min. These facts suggest that the anomalous temperature‐dependent UC emissions cannot be ascribed to the persistent luminescence of these phosphors because excitation of the samples with a 976 nm laser does not lead to TL peaks. The recovery ability of the temperature‐dependent UC spectra of the samples also reveals that there is no TL effect in the temperature‐dependent UC processes. The temperature‐dependence of Cr^3+^ emission can be explained by the configurationally coordinate model, in which different thermal activation energies for each equilibrium excited state at different temperatures are considered. The Er^3+^ emission is ascribed to the complex forward and backward energy transfer processes among the dopants Cr^3+^/Yb^3+^/Er^3+^. The reason for the absence of UCPL needs to be investigated further.

**Figure 13:**
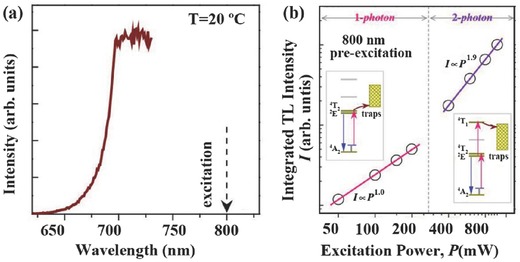
a) Photoluminescence emission spectrum of Zn_3_Ga_2_GeO_8_: Cr^3+^ under an 800 nm laser diode excitation at RT. b) Plot of the integrated TL intensities as a function of the excitation power. The left and right insets are the proposed one‐photon and two‐photon trap filling mechanisms corresponding to low‐ and high‐excitation powers, respectively. Reproduced with permission.^51^ Copyright 2016, Optical Society of America.

UC emission of Cr^3+^ could also be observed without a sensitiser, such as Ln^3+^. A sharp UC emission peak at ≈694 nm originating from ^2^ *E*→^4^ *A*_2_ of Cr^3+^ in the Al_2_O_3_ crystal without Yb^3+^ was observed when irradiated with an IR femtosecond laser at 800 nm.^50^ Because there is no energy level at ≈800 nm according to the absorption spectrum of Al_2_O_3_:Cr^3+^, a simultaneous two‐photon absorption mechanism is proposed for the UC process based on the ultrafast characteristics of the femtosecond laser. However, the same result can be achieved in Zn_3_Ga_2_GeO_8_: Cr^3+^ upon excitation with continuous‐wave 800 nm laser diodes with sufficient pumping power.^51^ As observed in **Figure** **13**, the broadband 650–730 nm emission of Cr^3+^, owing to the incorporation of the ^2^ *E*→^4^ *A*_2_ and ^4^ *T_2_*→^4^ *A*_2_ transitions, is observed with 800 nm laser excitation. This material also shows persistent luminescence of Cr^3+^, and the temperature‐dependent excitation photon energy and the persistent luminescence intensity suggest that it involves phonons during the excitation process. The pumping‐power‐dependent integrated TL intensity in Figure 13 indicates that it is a phonon‐assisted one‐photon process at low power and a two‐photon process at high power. It could fill low‐energy traps for the former and high‐energy traps for the latter. The unique single NIR emission band characteristic of Cr^3+^ may also be attractive for bioimaging applications.

### 2.4 Upconversion of Some Other TM Ions

**Figure 14:**
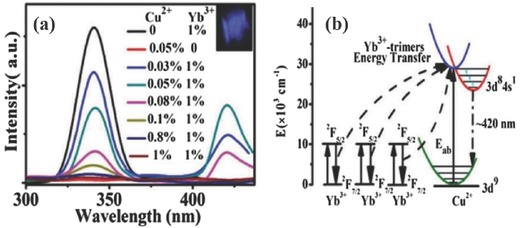
a) UC emission spectra (300–450 nm) of CaF_2_: *x*%Yb^3+^,*y*%Cu^2+^ upon excitation of 978 nm laser at RT. Inset is the photo of UC luminescence. b) Schematic diagram of the energy transfer from Yb^3+^‐trimer to one Cu^2+^. Reproduced with permission.^52^ Copyright 2016, The Royal Society of Chemistry.

There are few cases of other TM ions demonstrating UC emission at RT because they all have complex and abundant energy levels in the VIS and IR region. Qin. et al.^52^ recently reported that the UC emission of the Cu^2+^: 3*d* ^8^4*s* ^1^→3*d* ^9^ transition at ≈420 nm was observed in CaF_2_:Yb^3+^,Cu^2+^ upon 978 nm NIR laser excitation, as shown in **Figure** **14**, owing to the energy transfer process from the Yb^3+^ trimer to Cu^2+^ in the host CaF_2_; this process was indicated by the observation of triplet cooperative luminescence at ≈343 nm from the Yb^3+^ trimer and the UC decay curve variation of the Yb^3+^ trimer, Yb^3+^ dimer and Cu^2+^ emission. The splitting of the Cu^2+^ blue emission into three peaks at low temperature was due to the Jahn‐Teller effect of Cu^2+^ in CaF_2_. It is interesting that the 3*d* ^8^4*s* ^1^→3*d* ^9^ transition of Cu^2+^ is not quenched by its *d*–*d* transition and the *f*–*f* transition of the Ln^3+^ ion impurity introduced by the raw materials.

In addition to the sensitisation effect of Ln^3+^ in the UC process, TM ions, themselves with a suitable gap between energy levels, can also absorb the pumped light to facilitate the UC process. For instance, broadband green UC luminescence of Ni^2+^ in KZnF_3_ is observed at all temperatures ranging from 15 K to RT by excitation into the ^3^ *T*_1g_ (3*F*) or ^1^ *E*_g_ excited state of Ni^2+^ with monochromatic light, which is quite different from that of Ni^2+^‐doped chloride and bromide materials with the ^1^ *T*_2g_→^3^ *A*_2g_ luminescence of Ni^2+^ located in the red region and quenched well below RT.^53^ Normally, the overall UC efficiency is low in UC materials for narrowband laser excitation. However, an increase of roughly an order of magnitude is observed for Cs_2_NaYCl_6_:V^3+^, Re^4+^ compared with that of a Re^4+^ singly doped broadband excitation,^8^ which is attributed to the broadband absorption characteristics of TM ions. The UC luminescence of this material can be observed by the naked eye up to RT.

**Figure 15:**
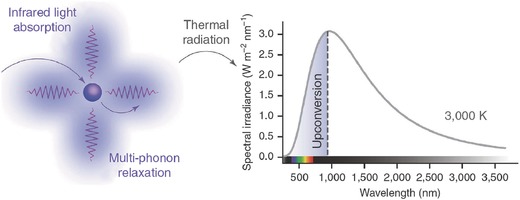
Schematic diagram illustrating photon energy upconversion by thermal radiation. Reproduced with permission.^54^ Copyright 2014, Macmillan Publishers Limited.

There is another distinct UC process of thermal radiation when sensitised by TM ions in Cu^2+^‐ or Cr^3+^‐doped ZrO_2_.^54^ This is a type of UC process that absorbs NIR sunlight or laser energy, resulting in a temperature increase of the material's body through multiphonon relaxation, followed by release via thermal radiation, such as the blackbody radiation, as illustrated in **Figure** **15**. The broadband absorption nature of Cu^2+^ and Cr^3+^ makes the Cu^2+^‐ or Cr^3+^‐doped ZrO_2_ sample glow when excited by concentrated and filtered sunlight, whereas the Yb^3+^‐doped ZrO_2_ sample, with a relatively narrow absorption band, does not glow. However, the latter exhibits the highest UC power efficiency when stimulated by laser, as high as 16% greater than the former. This is because the power of concentrated and filtered sunlight is smaller than the power of a laser. The authors also stated that TM dopants inevitably alter the thermal conductivity, melting point and refractive index of the host, especially at a high doping level. The Cu^2+^‐ or Cr^3+^‐doped ZrO_2_ samples may suffer high thermal conductivity. Therefore, the authors concluded that materials with higher melting points and lower thermal conductivities would work at higher blackbody temperatures and dissipate less heat with higher UC efficiency.^54^

## 3 Upconversion of Ln^3+^ Tuned by TM or *d* ^0^ Ions

### 3.1 Upconversion of Ln^3+^ Modulated by TM Ions

Doping with TM ions is a novel strategy for synthesising controllable UC nanomaterials in terms of nanocrystal growth and the resulting UC behaviour. For instance, Mn^2+^‐doping affects the growth dynamics and provides simultaneous control of the crystalline phase and the size of the resulting NaYF_4_:Yb/Er UC nanoparticles synthesised using a modified liquid‐solid solution solvothermal strategy.^55^ Without Mn^2+^ doping, the resulting product is a mixture of cubic and hexagonal phases. The hexagonal phase changes to the cubic phase after doping with a sufficient amount of Mn^2+^. Generally, dopant ions with larger ionic radii favour hexagonal structures, whereas smaller dopant ions tend to produce the cubic phase in the final products.^56^ In this case, the smaller Mn^2+^ ion incorporated into NaYF_4_ nanocrystals favours the formation of a pure cubic phase. However, Mn^2+^ substituted for Y^3+^ in NaYF_4_ can result in an extra F^‐^ ion on the grain surface and induces transient electric dipoles with their negative poles pointing outward. This effect would substantially hinder the diffusion of the F^‐^ ions required for crystal growth from the solution to the grain surface owing to charge repulsion, resulting in retardation of NaYF_4_ nanocrystal growth.^55^, ^56^ Similar phase transformation phenomena were observed in NaLnF_4_:Yb/Er (Ln = Lu, Yb, Gd) UC nanoparticles,^57^ with simultaneous phase/size control and significantly enhanced UC intensity via Mn^2+^ doping. However, a conflicting result, with uniform bundle‐shaped β‐NaYF_4_ (hexagonal phase) microtubes composed of six half‐pipes synthesised in hydrothermal solutions with trisodium citrate and Mn^2+^ doping, was obtained, which was proposed to be caused by an intentional delayed phase‐transition pathway induced by Mn^2+^.^58^ A further demonstration of small Mn^2+^‐modulated NaYF_4_ nanocrystals synthesised by hot‐injection was provided in a recent work,^59^ indicating that the Mn^2+^ dosage, growth temperature, and heating rate determine the doping modes (surface or interior). Interior doping contributes to red emission, whereas surface doping suppresses the growth of large nanocrystals.

**Figure 16:**
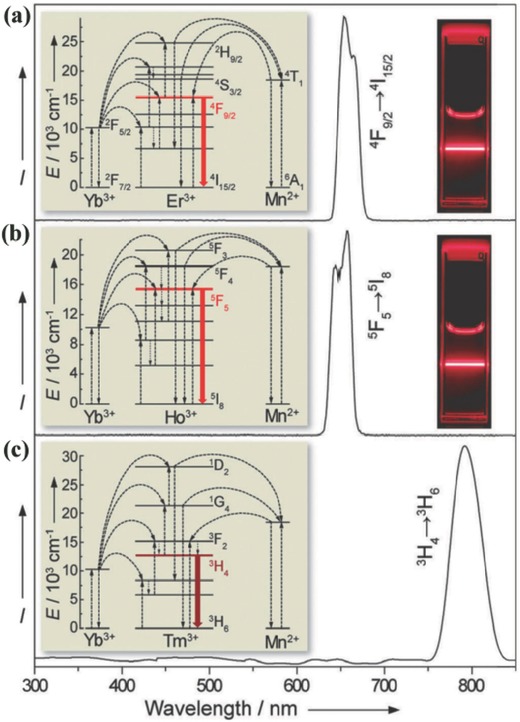
RT UC emission spectra of solutions containing: a) KMnF_3_:Yb/Er (18:2 mol %), b) KMnF_3_:Yb/Ho (18:2 mol %), and c) KMnF_3_:Yb/Tm (18:2 mol %) nanocrystals in cyclohexane (insets: proposed energy transfer mechanisms and corresponding luminescent photos of the colloidal solutions). All spectra were recorded under excitation of a 980 nm CW diode laser at a power density of 10 W cm^–2^. Reproduced with permission.^61^

**Figure 17:**
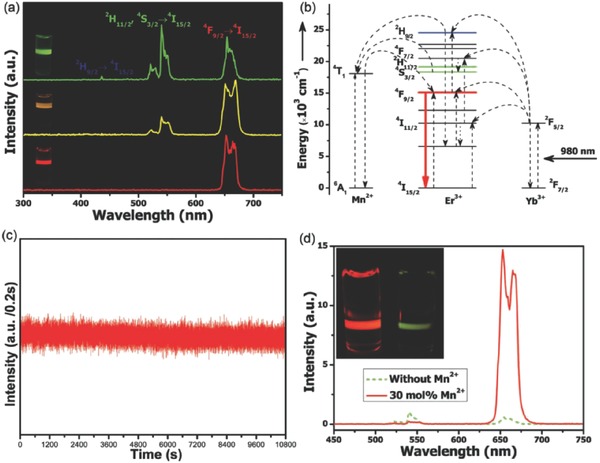
a) RT UC emission spectra of NaYF_4_: Yb/Er (18/2 mol %) nanocrystals with 0, 5 and 30 mol % Mn^2+^ dopant ions dispersed in cyclohexane (1 mg mL^–1^), respectively; inset: luminescent photographs of the corresponding samples. b) Schematic energy level diagram showing the possible UC mechanism of Mn^2+^‐doped NaYF_4_:Yb/Er (18/2 mol %) nanocrystals. c) The luminescence time traces of the 30 mol % Mn^2+^‐doped NaYF_4_:Yb/Er (18/2 mol %) UC nanoparticles acquired with 200 ms time bins under continuous 980 nm laser illumination for more than 3 h, suggesting the durable photostability of the UC nanoparticles. d) Comparison of RT UC emission spectra of NaYF_4_:Yb/Er (18/2 mol %) nanocrystals with 0 and 30 mol % Mn^2+^ dopant ions dispersed in cyclohexane (1 mg mL^–1^), respectively. Inset: the corresponding luminescent photographs. Reproduced with permission.^55^

Mn^2+^ is capable of tuning the UC behaviour of Ln^3+^ ions via Mn^2+^‐induced morphology/phase control and through the energy levels of Mn^2+^, such as in MnF_2_:Yb^3+^,Er^3+^.^60^ It was recently demonstrated that Mn^2+^ could tune the UC emission of Er^3+^, Ho^3+^, and Tm^3+^ into a single emission band in KMnF_3_ when codoping with Yb^3+^,^61^ as demonstrated in **Figure** **16**. Generally, these Ln^3+^ ions have more than one metastable excited state energy level, resulting in multiple emission peaks and low chromatic colour purity. The single‐band emissions of Er^3+^, Ho^3+^, and Tm^3+^ in KMnF_3_ are caused by the efficient forward and backward energy transfer between Er^3+^/Ho^3+^/Tm^3+^ and Mn^2+^ at different energy levels, as shown in Figure 16. In addition, the single‐band emission is independent of the dopant concentration, pump power, and temperature, which provides additional evidence of the efficient energy transfer pathway. The KMnF_3_:Er^3+^,Yb^3+^ prepared by an oil‐based synthetic procedure in this work has a higher ratio of red to green emission than that synthesised by the hydrothermal method in a previous work,^62^ likely because of the more homogeneous doping of the large Ln^3+^ content into the KMnF_3_ host in the former. The single emission band feature of UC nanocrystals has been developed for applications in anti‐counterfeiting, colour displays, and deep‐tissue imaging without constraints. Intense pure red emission is also obtained in sub‐10 nm NaMnF_3_:Yb^3+^,Er^3+^ nanocrystals.^19^ Analogous tuned behaviour of NaYF_4_:Yb^3+^,Er^3+^ nanoparticles by Mn^2+^ doping was observed in previous research,^55^, ^58^, ^59^ one of which is illustrated in **Figure** **17**. It is attributed to the non‐radiative energy transfer from the ^2^ *H*_9/2_ and ^4^ *S*_3/2_ levels of Er^3+^ to the ^4^ *T*_1_ level of Mn^2+^, followed by back energy transfer to the ^4^ *F*_9/2_ level of Er^3+^, as discussed above. Mn^2+^ doping facilitates the single band feature of red emission and enhances the intensity of UC emission, which arises from the change in the surrounding environment of Ln^3+^ ions and the energy transfer between Er^3+^ and Mn^2+^ ions. Water‐soluble and biocompatible poly(ethylene glycol)‐conjugated phospholipid (DSPE‐PEG 2000) is used to coat the oleate‐capped nanocrystals for imaging of deep tissue in Kunming mouse. The modified nanocrystal solutions are injected respectively at foot, back and upper leg regions of the mice to investigate the dose effect. The injection depth is estimated by the needle penetration (about 10 mm). Results show that the signals from such deep tissue are obvious for the Mn^2+^ doped samples. The Mn^2+^ doping also benefit the magnetic resonance imaging as a second imaging capacity.^55^ Additionally, significant tuning of the output colour by adjusting the contents of Mn^2+^ is only obtained in cubic NaYF_4_ nanoparticles and not in hexagonal microtubes.^58^ Tian et al.^63^ proposed that the incorporation of Mn^2+^ in hexagonal NaYbF_4_:Er reduced the Na vacancies to offset the imbalance of charge and reduced organic absorption on the surface of the nanocrystals by the extra F^–^ and Mn^2+^ on the surface, which resulted in enhanced UC emission of Er^3+^. There is additional research work on Mn^2+^ incorporated in fluorides, such as in the new host matrix KLu_3_F_10_:Yb,Mn,Er/Ho/Tm,^64^ the plasma coupling effects of an Ag array on NaYF_4_:Yb,Er,Mn,^65^ and the high magnetic field and temperature tuning effects on NaYF_4_:Yb,Er,Mn.^66^

**Figure 18:**
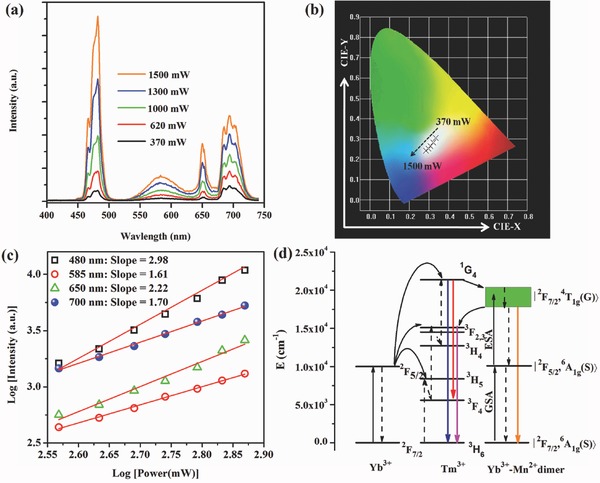
a) Pump‐power dependence of UC emission spectra, b) CIE chromaticity coordinates, and c) double‐logarithmic plots of the pump‐power dependent UC emission intensity of KZnF_3_:1%Yb^3+^,0.1%Tm^3+^,1%Mn^2+^; d) the sketch of related UC processes. Reproduced with permission.^67^ Copyright 2014, Optical Society of America.

As observed above, Mn^2+^ emission is rarely observed when codoped with Ln^3+^ in an Yb^3+^–Mn^2+^ UC system owing to the complex forward and backward energy transfer between Ln^3+^ and Mn^2+^ ions,^55^, ^57^, ^60^, ^61^ which have abundant energy levels above and below the ^4^ *T*_1_ emitting state of Mn^2+^. There are two approaches to obtain additional Mn^2+^ emission besides the Ln^3+^ emission in these triply codoped systems. One is to decrease the amount of Ln^3+^;^67^, ^68^ the other is to control the spatial distribution of Ln^3+^ and Mn^2+^ with a certain separated distance, such as using a core‐shell structure.^69^ For the combination of Mn^2+^ emission with Ln^3+^ emission, white light can be easily achieved, as in the example of KZnF_3_:Yb^3+^,Mn^2+^,Tm^3+^ in **Figure** **18**.^67^ The sharp emission peaks at 480, 650 and 700 nm in Figure 18a can be ascribed to the ^1^ *G*_4_→^3^ *H*_6_, ^1^ *G*_4_→^3^ *F*_4_, and ^3^ *F*_2,3_→^3^ *H*_6_ transitions of the Tm^3+^ ion, respectively. The broad emission band centred at 585 nm corresponds to the emission of Mn^2+^. The CIE chromaticity coordinates of white light shift slightly with variation of the pumping power, as shown in Figure 18b, which is due to the complex energy transfer involved in Yb^3+^/Mn^2+^/Tm^3+^. The power dependency of the UC emission intensities with different slope values in Figure 18c suggest that the 480 and 650 nm UC emissions are three‐photon processes, whereas the 585 and 700 nm UC emissions are two‐photon processes. The ^4^ *T*_1g_ excited state of Mn^2+^ is lower than the ^1^ *G*_4_ excited state of Tm^3+^ but higher than the ^3^ *F*_2,3_ excited state of Tm^3+^. Therefore, energy transfer between Mn^2+^ and Tm^3+^ ions may occur in Yb^3+^/Tm^3+^/Mn^2+^ tri‐doped KZnF_3_, in which the energy is transferred from ^1^ *G*_4_ of Tm^3+^ to ^4^ *T*_1g_ of Mn^2+^ and then back to the ^3^ *F*_2,3_ state of Tm^3+^. A possible UC mechanism in the Yb^3+^/Tm^3+^/Mn^2+^ tri‐doped system is proposed in Figure 18d. The broadband yellow UC luminescence is resulted from sequential GSA/ESA processes for the Yb^3+^–Mn^2+^ dimer. The 700 nm band of Tm^3+^ was enhanced by bi‐directional energy transfer between Tm^3+^ and the Yb^3+^–Mn^2+^ dimer.

**Figure 19:**
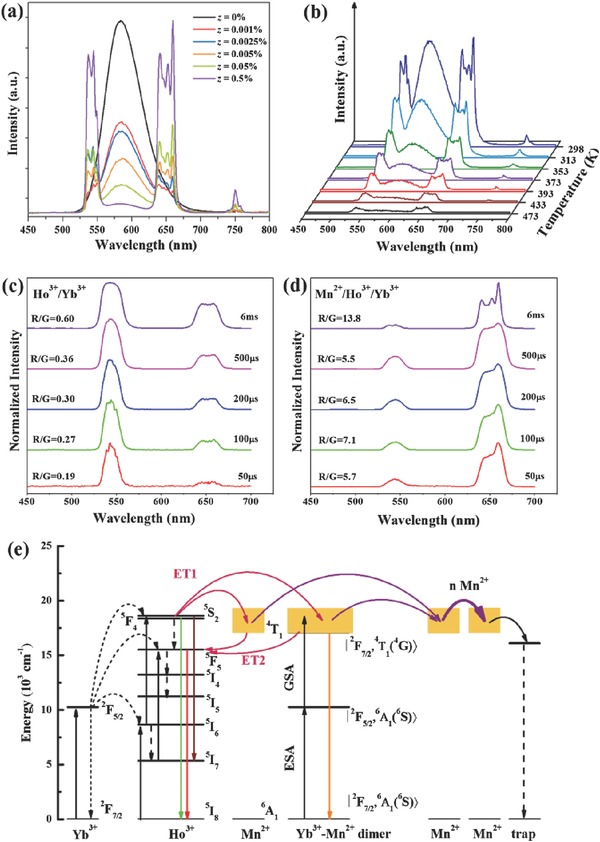
a) UC emission spectra of KZnF_3_: 5%Mn^2+^, 1%Yb^3+^, *z*Ho^3+^ under 976 nm LD excitation; b) Temperature‐dependent UC emission spectra of KZnF_3_: 5%Mn^2+^, 1%Yb^3+^, 0.005%Ho^3+^; UC emission spectra of the (c) KZnF_3_: 0.5%Yb^3+^,1%Ho^3+^ and (d) KZnF_3_: 15%Mn^2+^,0.5%Yb^3+^,1%Ho^3+^under 976 nm LD excitation with different pulse duration; e) Schematic illustration of possible UC processes. Reproduced with permission.^68^ Copyright 2016, Elsevier B.V.

The concentration‐dependent, temperature‐dependent and laser‐pulse‐duration‐dependent energy transfer processes among Yb^3+^/Ho^3+^/Mn^2+^ ions in KZnF_3_ were investigated in detail, and some of the results are presented in **Figure** **19**.^68^ Upon 976 nm laser excitation, the KZnF_3_:Yb^3+^,Mn^2+^ sample exhibits a broad UC emission band centred at 585 nm in Figure 19a, originating from the |^2^ *F*_7/2_,^4^ *T*_1_>→ |^2^ *F*_7/2_,^6^ *A*_1_> transition of Yb^3+^–Mn^2+^ dimers. Three typical emission peaks located at 539, 658 and 749 nm appeared with the introduction of Ho^3+^ ions into KZnF_3_:Yb^3+^,Mn^2+^ and are ascribed to the ^5^ *S*_2_/^5^ *F*_4_→^5^ *I*_8_, ^5^ *F*_5_→^5^ *I*_8_ and ^5^ *S*_2_/^5^ *F*_4_→^5^ *I*_7_ transitions of Ho^3+^, respectively. The yellow UC emission from Yb^3+^–Mn^2+^ dimers sharply decreases and is nearly quenched as the concentration of Ho^3+^ increases to *z* = 0.5%. Meanwhile, the green and red UC emissions from Ho^3+^ are enhanced. For the KZnF_3_: 1%Yb^3+^,0.005%Ho^3+^,5%Mn^2+^ sample, the temperature‐dependent emission intensity of all UC bands in Figure 19b decrease monotonically with increasing temperature, which is similar to that of undoped samples and is due to the non‐radiative multiphonon process. The ratio of R/G behaves distinctively. When the temperature is between 298 and 373 K, the ratio of R/G gradually decreases from 1.36 to 0.98 with increasing temperature (not shown in Figure 19). Further increased the temperature increases the R/G ratio. The decrease in the R/G ratio between 298 and 373 K suggests that there is a new process to be considered. The strong red UC emission of Ho^3+^ in this system is due to the bidirectional energy transfer (ET1 and ET2) between Ho^3+^ and Mn^2+^ (Yb^3+^–Mn^2+^ dimer), as shown in Figure 19e. Because of ET1, some of the energy of Ho^3+^ can be transferred to the ^4^ *T*_1_ state of Mn^2+^ or the |^2^ *F*_7/2_,^4^ *T*_1_> state of the Yb^3+^–Mn^2+^ dimer. With increasing temperature, the non‐radiative relaxation of Mn^2+^ or Yb^3+^–Mn^2+^ dimer is inevitable, promoting the dissipation of energy by defects or through the surface, which hinders the back energy transfer (ET2) to the ^5^ *F*_5_ state and leads to a sharply decreasing ratio of R/G UC emission with increasing temperature. When the temperature exceeds 373 K, the multiphonon relaxation from ^5^ *I*_6_ to ^5^ *I*_7_ dominates, as it does in the Ho^3+^/Yb^3+^‐codoped system, resulting in the increase. Figure 19c and Figure 19d show the UC emission spectra of KZnF_3_:1%Yb^3+^,1%Ho^3+^ and KZnF_3_:15%Mn^2+^,1%Yb^3+^,1%Ho^3+^ under excitation with a 976 nm laser with different pulse widths (50 µs ≈ 6 ms). When KZnF_3_:1%Yb^3+^,1%Ho^3+^ is pumped by a long‐pulse‐width laser (6 ms, 100 Hz), the characteristic intense green and red UC emissions of Ho^3+^ are observed. The UC spectrum under long‐pulse‐width laser excitation is similar to that under continuous‐wave laser excitation, indicating that the long pulse width (6 ms, 10 Hz) provides a steady‐state upconversion process for Ho^3+^. Decreasing the excitation pulse width from 6 ms to 50 µs decreases the R/G from 0.6 to 0.19, which could be explained by the different population processes of the excited states leading to 539 and 658 nm emission. When KZnF_3_:15%Mn^2+^,1%Yb^3+^,1%Ho^3+^ is excited by a laser pulse width from 500 µs to 50 µs, the R/G ratio is nearly unchanged and the UC emission spectra are still dominated by red UC emission. R/G increases to 13.8 with increasing the pulse width from 500 µs to 6 ms owing to the different cross relaxation rates between Yb^3+^ and Ho^3+^ and the Mn^2+^–Ho^3+^ energy transfer. The red emission level could be further populated by the ^5^ *I*_7_ state with long excitation duration owing to the long lifetime of the ^5^ *I*_7_ intermediate state (4 ms). Increasing pulse width allows for a greater possibility of cross relaxation between Yb^3+^ and Ho^3+^, providing an additional population path for red UC emission. Generally, there are energy levels below ≈10,000 cm^–1^ for Tm^3+^, Ho^3+^ and Er^3+^ ions. Therefore, there is inevitably NIR emission when pumping with a 980 nm laser in these Yb^3+^–Ln^3+^–Mn^2+^ systems. The green UC emission at ≈533 nm and the NIR emission at ≈1500 nm of Er^3+^ could be selectively enhanced by codoping Mn^2+^ in MgGa_2_O_4_:Yb^3+^,Er^3+^ owing to the sensitisation of the Yb^3+^‐Mn^2+^ dimer.^70^

**Figure 20:**
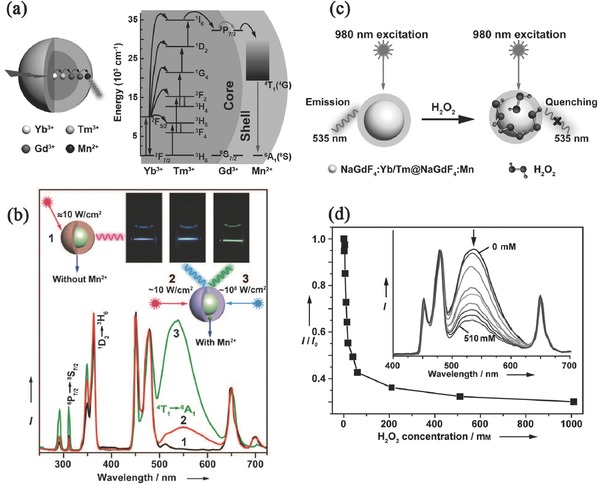
a) Illustration of NaGdF_4_:Yb/Tm@NaGdF_4_:Mn core‐shell structure and the proposed energy transfer pathway; b) UC emission profiles of 1) NaGdF_4_:Yb/Tm@NaGdF_4_ and 2) NaGdF_4_:Yb/Tm@NaGdF_4_:Mn at RT upon 980 nm CW excitation as well as 3) NaGdF_4_:Yb/Tm@NaGdF_4_:Mn nanostructure pumped by a pulsed OPO laser. Insert are the photos of cyclohexane solutions of the nanoparticles; c) Schematic illustration of H_2_O_2_ sensing; d) Emission intensity dependence (as measured by the ratio of *I*/*I*_0_ at 535 nm) on H_2_O_2_ concentration, inset is the corresponding UC emission spectra. Reproduced with permission.^69^

Liu et al.^69^ synthesised the core‐shell NaGdF_4_:Yb/Tm@NaGdF_4_:Mn nanostructure, which produced the 535 nm emission of Mn^2+^, as observed in **Figure** **20**. Five‐photon UC processes populate the ^1^ *I*_6_ state of Tm^3+^ ions; then, energy migrates through the Gd^3+^ sublattice at its ^6^ *P*_7/2_ state, followed by energy transfer to Mn^2+^, giving the ^4^ *T*_1_→^6^ *A*_1_ emission. EXAFS suggested that the average first‐shell Mn–F coordination number for Mn^2+^ ions is approximately 6, which is lower than that of Gd^3+^ ions (≈8) for first‐shell Gd–F in cubic NaGdF_4_. This may be caused by the formation of F^–^ vacancies in the crystal lattice to compensate for the charge imbalance when Gd^3+^ ions are substituted by Mn^2+^ ions. DFT calculation of the formation energy revealed that replacement of Gd^3+^ with Mn^2+^ requires less energy than the substitution of Na^+^ by Mn^2+^ ions. Moreover, introducing F^–^ vacancies in the model of Mn^2+^‐substituted Gd^3+^ decreases the formation energy, which is consistent with the EXAFS results. Owing to the strong tendency for Mn^2+^ ions to undergo oxidation, the core‐shell structure can be used as a sensing probe for H_2_O_2_ molecules, as shown in Figure 20c,d. The intensity of Mn^2+^ UC emission decreases gradually with increasing H_2_O_2_ content, while that of Tm^3+^ varies little. The oxidizable nature of Mn^2+^ in the Mn^2+^‐doped UC nanocrystals may make it to be a promising luminescent probe for real‐time monitoring H_2_O_2_ generation in a variety of biological processes.^69^

The green and red UC emissions of Er^3+^ in β‐NaYF_4_:Yb^3+^,Er^3+^ nanocrystals can be enhanced by codoping with Fe^3+^.^71^ Doping with Fe^3+^ ions does not result in apparent impurity phases, even at a high doping concentration of 40%, as reported in this work. A possible mechanism for the enhanced UC emissions proposed by the authors is that Fe^3+^ dopants lower the symmetry of the environment around Er^3+^, which increases the probability of an *f*–*f* electric dipole transition of Er^3+^. However, the mechanism requires further investigation because Fe^3+^ has abundant energy levels, which normally leads to quenching of the visible emission.

### 3.2 Upconversion of Ln^3+^ Ions Sensitised by TM Ions

**Figure 21:**
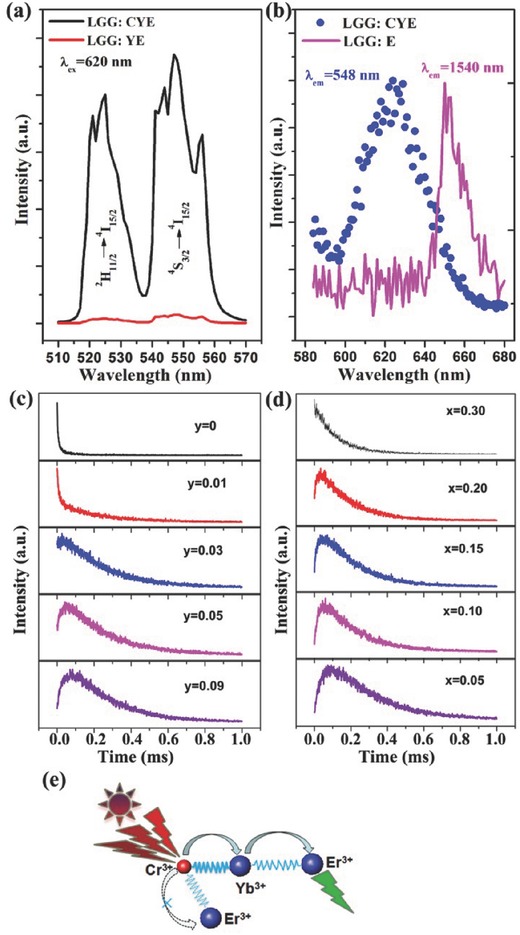
a) UC luminescence spectra of LGG:CYE(short for La_2.82_Ga_4.95_GeO_14_: 0.05Cr^3+^, 0.12Yb^3+^, 0.06Er^3+^) and LGG:YE (short for La_2.82_Ga_5_GeO_14_:0.12Yb^3+^, 0.06Er^3+^) pumped by an OPO pulsed laser(620 nm) with the same power density(about 50 mW mm^–2^); b) the monitored 548 nm UC emission intensity pumped by different wavelengths(blue filled circle); for comparison, excitation peak of LGG:E(short for La_2.94_Ga_5_GeO_14_:0.06Er^3+^) at around 650 nm is also demonstrated(magenta line); UC luminescence decay curves of LGG: 0.10Cr^3+^, yYb^3+^, 0.06Er^3+^ (c) and LGG: xCr^3+^, 0.12Yb^3+^, 0.06Er^3+^ (d) pumped by an OPO pulsed laser (λ_ex_ = 620 nm, λ_em_ = 548 nm); e) Illustration of energy transfer pathway in the UC process in this system. Reproduced with permission.^75^ Copyright 2014, OSA.

A sensitisation process is normally required to improve the performance of UC materials,^6^, ^8^, ^12^, ^13^, ^72^, ^73^, ^74^, ^75^ and there is room for creativity. Dye molecules with broadband absorption characteristic can efficiently sensitise the UC luminescence of β‐NaYF_4_:Er^3+^,Yb^3+^,^73^ which provides upconverted emission from Ln^3+^ ions under broadband low‐power excitation. Broadband sensitisation is currently a hot topic in the research of UC materials,^74^ which is motivated by the fact that UC materials suffer from a high pumping energy density and an arrow Ln^3+^ excitation line. TM ions exhibit broadband absorption, which is commonly utilised to sensitise the Stokes luminescence of Ln^3+^.^12^ Analogously, TM ions can also be used as broadband sensitisers for UC luminescence.^8^, ^12^, ^13^ Piguet et al.^11^ reported and discussed the very inefficient UC process in Ln^3+^ supramolecular complexes, in which organic molecule ligands cause strong multiphonon relaxation between energy levels in Ln^3+^ because of the large phonon energy of approximately 2000 cm^–1^ of the organic molecules. This would result in shortening of the metastable excited state lifetime of Ln^3+^ and make the UC of Ln^3+^ via the ESA mechanism too inefficient to be detected under practical excitation intensities, even at cryogenic temperatures. Theoretically, they proposed that the combination of two TM ions as sensitisers with an Ln^3+^ activator in polynuclear *d*‐*f*‐*d* supramolecular complexes would overcome the limitation via the ETU process. The authors experimentally obtained UC emission at 33 K.^11^ However, it is rare for TM–Ln^3+^ ion codoped systems to exhibit RT UC luminescence because the non‐radiative relaxation rate of TM ions is generally large, resulting in depopulation of the intermediate energy level of the TM ions serving as a storage level for subsequent UC processes. It is preferable to design TM ions as sensitisers for the long‐lived intermediate excited energy level of Ln^3+^ ions in which the UC process occurs. In this manner, the TM‐sensitised UC luminescence of Yb^3+^–Er^3+^, which is considered as a high‐efficiency UC luminescence system, may be obtained at RT. Experience reveals that the material host with the desired RT UC luminescence should contain proper sites for accommodating the TM and Ln^3+^ ions and that the TM ions should not absorb the desired emission of Ln^3+^.^6^, ^10^, ^38^ According to this idea, the broadband light management phenomenon in the UC material La_3_Ga_5_GeO_14_:Cr^3+^,Yb^3+^,Er^3+^ has been observed at RT (see **Figure** **21**a and 21b),^75^ which is ascribed to the absorption of Cr^3+^. Energy transfer among Cr^3+^/Yb^3+^/Er^3+^ in the Stokes and UC luminescence processes is discussed in detail in this research work. As observed in Figure 21c, UC luminescence of Er^3+^sensitised by Cr^3+^ directly with 620 nm pulsed laser light is inefficient because almost no decay signal of Er^3+^ is observed without Yb^3+^. As the Yb^3+^ content increases, the decay curves prolong, which can be interpreted as energy transfer from Cr^3+^ to Er^3+^ through Yb^3+^ as a “bridge” in the UC process. The decay curves decline with increasing Cr^3+^ contents in Figure 21d, suggesting large contents of Cr^3+^ interfere with the energy transfer from Cr^3+^ to Yb^3+^–Er^3+^. Figure 21e schematically illustrates the energy transfer processes. An obvious increase in the early stage of the decay curves occurs with the increase of the Yb^3+^ contents, indicating that the UC mechanism is ETU. Therefore, the designed UC emissions ^2^ *H*_11/2_→^4^ *I*_15/2_ and ^4^ *S*_3/2_→^4^ *I*_15/2_ of Er^3+^ at approximately 510–560 nm are proposed to occur through the ETU process based on the Cr^3+^–Yb^3+^ dimer model with superexchange interactions according to the crystallographic data. The material is also excitable by concentrated broadband noncoherent simulated sunlight, which would largely benefit the application of such materials in solar cells.

**Figure 22:**
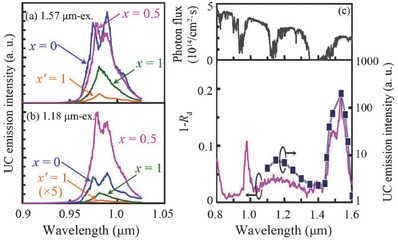
UC emission spectra of the 10% Er, 0.5% Ni, and 0.5% Nb‐doped LaGa_1–_ *_x_*Sc*_x_*O_3_ (*x* = 0, 0.5, and 1) and LaInO_3_ (*x*' = 1) powder samples excited at (a) 1570 nm and (b) 1180 nm; (c) Comparison of the absorbance (1–R_d_) and UC sensitivity of the sample with x = 0.5 (lower), the AM1.5G solar spectrum (upper) is also shown for reference. Reproduced with permission.^76^ Copyright 2016, AIP Publishing LLC.

Another case of broadband sensitised UC in oxide compounds at RT is La(Ga_0.5_Sc_0.5_)O_3_:Er^3+^,Ni^2+^,Nb^5+^, which converts 1100–1350 nm and 1450–1580 nm photons to 980 nm photons for application in crystalline silicon solar cells.^76^ The criteria for designing the host are similar to the La_3_Ga_5_GeO_14_:Cr^3+^,Yb^3+^,Er^3+^ case above, i.e., an La^3+^ site to accommodate the UC emission centre Er^3+^, and a Ga/Sc site with proper crystal field strength for the sensitiser Ni^2+^ to have long‐wavelength absorption band but no absorption band in the UC emission wavelength range. The NIR emission of Er^3+^ at ≈980 nm can be obtained by exciting either Er^3+^ at 1570 nm or Ni^2+^ at 1180 nm, as observed in **Figure** **22**a and 22b. The wavelength‐dependent UC sensitivity (similar to the excitation spectrum) in Figure 22c (bottom) reveals the broadband sensitisation characteristics at 1100–1350 nm and 1450–1580 nm, which are ascribed to the absorption of Ni^2+^ and Er^3+^, respectively. These absorption bands fit the solar energy flux in Figure 22c (top). This research facilitates the design of novel photonic materials that are excitable by broadband noncoherent light for applications such as solar cells.

### 3.3 Upconversion of Ln^3+^ Ions Tuned by *d* ^0^ Ions

**Figure 23:**
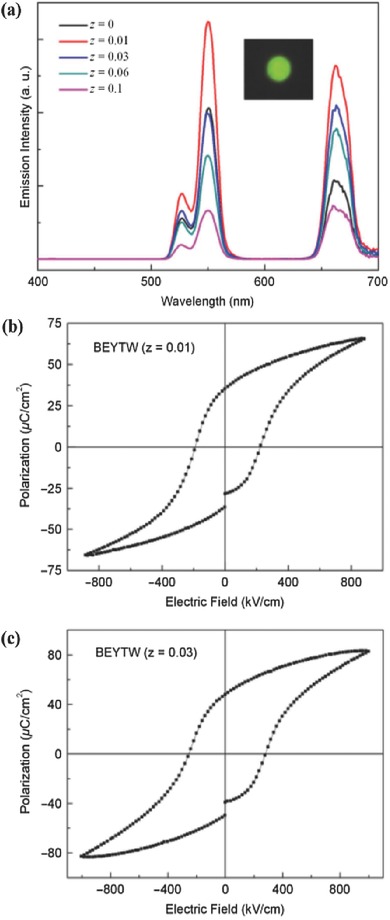
a) UC luminescence spectra of Bi_3.79_Er_0.03_Yb_0.18_Ti_3–_ *_z_*W*_z_*O_12_ thin films pumped by 980 nm laser. The inset shows the fluorescence photograph of Bi_3.79_Er_0.03_Yb_0.18_Ti_2.99_W_0.01_O_12_ thin films taken by a common digital camera. P‐E hysteresis loops of Bi_3.79_Er_0.03_Yb_0.18_Ti_3–_ *_z_*W*_z_*O_12_ thin films with W^6+^ ion contents of (b) *z* = 0.01 and (c) *z* = 0.03, respectively. Reproduced with permission.^78^ Copyright 2011, The American Ceramic Society.

The tuning effects of the Ln^3+^‐centred UC behaviour by *d* ^0^ ions are mostly ascribed to the electric diversity of materials with *d* ^0^ ion‐centred anion groups (including MoO_6_^6–^ and TiO_4_^4–^, etc.),^77^ which is based on the fact that the electric dipole transitions of Ln^3+^ ions are mostly affected by the surrounding electric environment comprised of *d* ^0^ ion‐centred anion groups. For example, the UC emission of Yb^3+^–Er^3+^ (the ratio of green to red emission) can be tuned by codoping W^6+^ in the ferroelectric material Bi_4_Ti_3_O_12_,^78^ as illustrated in **Figure** **23**. Ferroelectric compounds have a large dielectric permittivity, which is strongly correlated to the separation of the positive charge centre Ti^4+^ and the negative charge centre of the coordinated polyhedral O^2–^ in the *d* ^0^ ion‐centred anion group TiO_6_^8–^. The tuning mechanism may be that a larger polarisation effect would be induced on Er^3+^ by W^6+^ when substituting Ti^4+^, which might be evidenced by the larger polarisation value (see Figure 23) for the sample with higher W^6+^ content. This type of material has potential applications in multifunctional optoelectronic integrated devices.

**Figure 24:**
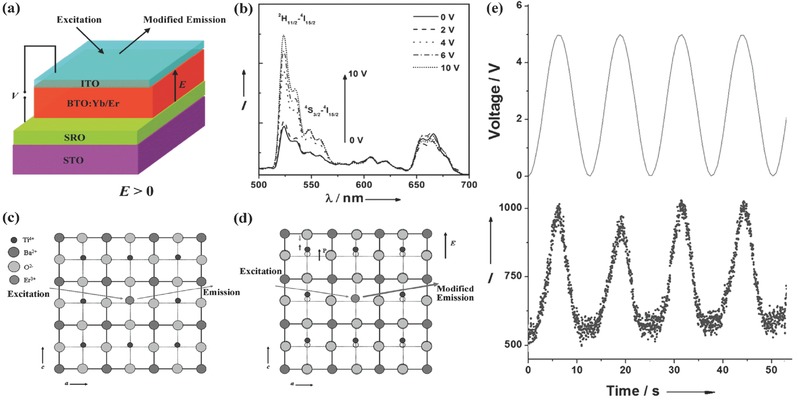
a) The setup used to measure the UC emission of a BaTiO_3_:Yb^3+^, Er^3+^ thin film when an external electric field is switched “on”. A 980 nm diode laser is used as an excitation source. b) The UC emission spectra of the thin film under direct current bias voltage ranging from 0 to 10 V; Schematic illustration of UC luminescence in the thin film lattice (c) without and (d) with external electric field *E*; e) Sinusoidal AC electric voltage applied to the film (top), and the resulting 523 nm emission as a function of time. Reproduced with permission.^16^

Direct evidence of the tuning behaviour of Yb^3+^–Er^3+^ in BaTiO_3_ ferroelectric thin films is achieved via an external electric field according to Hao,^16^ as illustrated in **Figure** **24**. The unique crystal structure of this ferroelectric host provides an opportunity to couple the variables, including the electric field, to the crystal symmetry, in addition to varying the chemical composition and/or doping, all of which affect UC behaviour.^16^ In the presence of an external electric field under low bias voltage, Ti^4+^ and O^2–^ in the lattice move in opposite directions, as shown in Figure 24d, resulting in the lower symmetry of the Er^3+^ site (i.e., internal electric field variation according to crystal field theory, which is based on the static point charge model) and enhancement of the Er^3+^ UC green emission by a factor of 2.7. The mechanism can be explained by Judd‐Ofelt theory for the electric dipole transition of the Er^3+^ ion. The green emission of Er^3+^ involves a hypersensitive transition, which is dominated by the Judd‐Ofelt intensity parameter *Ω*_2_. *Ω*_2_ is strongly associated with the asymmetry of the Ln^3+^ sites. Lower symmetry normally contributes to a larger *Ω*_2_, resulting in selective enhancement of the green emission of the Er^3+^ ion. Dynamic modulation of the UC emission of Er^3+^ is observed through in situ and real‐time periodical variation of the external electric field in Figure 24e, which suggests that the material could be used as electric controlled upconvertors.^16^ This research work provides a reversible and in situ approach to tune the UC behaviour, and the coupling of UC luminescence and the electric field opens new opportunities to design multifunctional materials and devices. The temperature‐induced internal electric field variation on Er^3+^ in Er^3+^‐doped perovskite PbTiO_3_ nanofibres can also tune the UC of Er^3+^ but in a contrasting way.^79^ The decrease of tetragonality and spontaneous polarisation of the nanofibres from 50 K to 300 K, revealed by in situ X‐ray diffraction, results in enhanced UC emission intensity of Er^3+^ for both the green and red emission, which is caused by the recovery of Ti^4+^ and O^2–^ to the equilibrium position for TiO_6_^8–^ octahedra with less distortion.^79^

Because the dielectric permittivity can reveal polarisation, effort was made to correlate the dielectric permittivity with the UC behaviour of Ln^3+^ in the Ti^4+^ based ferroelectric oxides. In a filled tetragonal tungsten bronze oxide Sr_4_(La_0.85_Ho_0.025_Yb_0.125_)_2_Ti_4_Nb_6_O_30_ ceramic, the Sr‐sites ionic occupation and distribution have a major influence on the local lattice distortion, whereas the dielectric permittivity may be a relatively sensitive probe of the local lattice distorted structure. Thus, it was expected that the UC variation of the Ho^3+^ ion was related to the dielectric permittivity in this Sr_4_(La_0.85_Ho_0.025_Yb_0.125_)_2_Ti_4_Nb_6_O_30_ ceramic when Sr^2+^ ion was substituted by Ba^2+^ ion.^80^ The variation tendency of the ^5^ *S*_2_→^5^ *I*_8_, ^5^ *F*_5_→^5^ *I*_8_ and ^5^ *S*_2_→^5^ *I*_7_ transition intensities of Ho^3+^ as a function of the Ba^2+^ content in this (Sr_1–_ *_x_*Ba*_x_*)_4_(La_0.85_Ho_0.025_Yb_0.125_)_2_Ti_4_Nb_6_O_30_ system is analogous to the dielectric permittivity under different frequencies of alternating current (*AC*) impedance spectroscopy, suggesting a correlation between the UC behaviour and the dielectric permittivity.^80^ It also suggests that both properties share the same structural origins. Dielectric permittivity is contributed by different types of polarisations (i.e., electronic, ionic, dipolar, and space charge). For the frequency range of 10 kHz–1.0 MHz, dielectric permittivity is mainly contributed by dipolar polarisation in response to an *AC* electric field. The random stress field caused by substituting Sr^2+^ with Ba^2+^ affects the octahedral (Ti/Nb)O_6_ and suppresses the Ti/Nb dipolar ion orientation along the *AC* electric field, resulting in reduced dielectric permittivity.^80^ The authors also stated^80^ that for *x* = 0 and *x* = 1.0, no random stress field existed; thus, high UC intensities were observed owing to the ordered Sr and Ba‐site alignment. For partial substitution cases, the random stress field was gradually enhanced with increasing *x*, and then suppressed when *x* approached 1.0. The random stress field induced by tilting and rotation of the (Ti/Nb)O_6_ octahedra around the Ho^3+^ site caused variation in the UC emission intensity.

However, the UC behaviour of Er^3+^ (ratio of red emission intensity to green emission intensity) had no direct relation with the dielectric, piezoelectric and ferroelectric properties in (0.97–*x*)Pb(Mg_1/3_Nb_2/3_)O_3_–*x*PbTiO_3_–0.03Pb(Er_1/2_Nb_1/2_)O_3_ ceramics but was correlated with the phase transition as *x* increased because there were three phases in the range of 0 < *x* < 0.40.^81^ Additional work^82^ showed that not all the electric dipole transitions of Ln^3+^ were sensitive to polarisation induced by the distortion of *d* ^0^ ion‐centred anion groups. The model of (Ba_0.77_Ca_0.23_)TiO_3_: Pr^3+^ was chosen to study the correlation because it showed the highest remanent polarisation and electrostrictive strain. As a result, a strong poling effect was expected, according to Zhang et al.^82^ The temperature (5–450 K) dependence of Pr^3+^ emission in this (Ba_0.77_Ca_0.23_)_1–_ *_x_*Pr*_x_*TiO_3_ ceramic was investigated. The blue emission (≈490 nm, ^3^ *P*_0_→^3^ *H*_4_) of Pr^3+^ has a strong thermal quenching effect, which disappears as the temperature increases to approximately 200 K, whereas the red emission (≈611 nm, ^1^ *D*_2_→^3^ *H*_4_) is resistant to thermal quenching, even above room temperature, partially because of the phase transition at 80–170 K. The poled sample (with a remanent polarisation of approximately 10 μC cm^–2^) shows 30% higher red emission intensity than that of the unpoled sample, whereas for the poled and unpoled Pr^3+^‐doped CaTiO_3_ or SrTiO_3_ ceramics without ferroelectricity, the red emission intensities change little. Therefore, the ferroelectric polarisation of the ceramics owing to the distortion of the *d* ^0^ ion‐centred anion group TiO_4_^4–^ could tune the red emission of Pr^3+^ but has little effect on the blue emission.^82^

**Figure 25:**
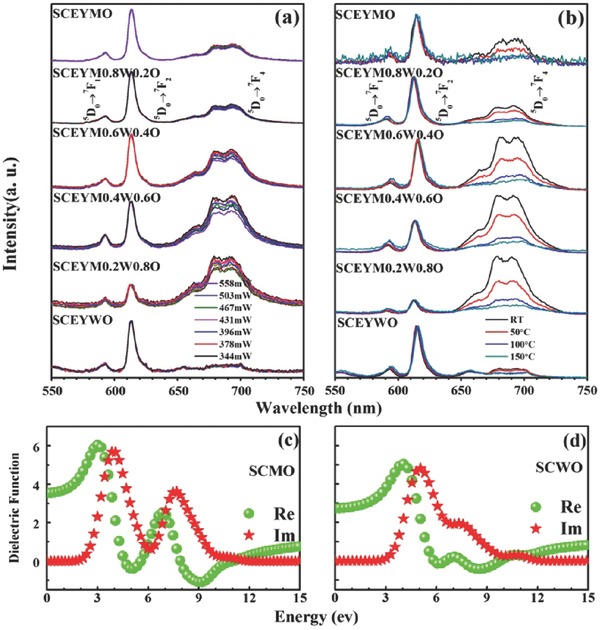
UC emission spectra of SCEYM_1–_ *_x_*W*_x_*O under the excitation of a 976 nm laser beam (a) with different pumping powers and (b) at different temperatures (all the spectra are normalized for the peaks at ≈612 nm corresponding to forced electric dipole transition of ^5^ *D*_0_→^7^ *F*_2_); Calculated imaginary parts (Im, red stars) and real parts (Re, green balls) of dielectric function for (c) SCMO and (d) SCWO compounds. Reproduced with permission.^84^ Copyright 2015, The Royal Society of Chemistry.

Another example of the selective influence of *d* ^0^‐centred anion groups on the electric dipole transition of Eu^3+^ was reported in Yb^3+^–Eu^3+^‐doped Sr_2_Ca(Mo,W)O_6_ double perovskites.^83^, ^84^, ^85^ A remarkable phenomenon, with an emission peak at ≈690 nm ascribed to the ^5^ *D*_0_→^7^ *F*_4_ transition of Eu^3+^, is observed in the UC spectra for the samples with Mo, whereas it is almost absent in the Stokes luminescence spectra. Furthermore, this peak almost disappears for samples without Mo in the UC spectra. Chan argued that this UC emission peak at ≈690 nm originated from the Mn^4+^ ions introduced by the MoO_3_ raw material.^86^ However, the emission of Mn^4+^ is excluded according to the most recent research work.^84^ According to Judd‐Ofelt theory, the laser‐beam‐induced polarisation effect of the MoO_6_^6–^‐group‐containing material may be responsible for the change in the ratio between the ^5^ *D*_0_→^7^ *F*_2_ and ^5^ *D*_0_→^7^ *F*_4_ electric dipole transitions when comparing samples stimulated by UV light and a 976 nm laser beam, which corresponds to the ligand polarisability‐dependent dynamic coupling mechanism. Further investigation of this anomalous UC emission of the ^5^ *D*_0_→^7^ *F*_4_ electric dipole transition of Eu^3+^ ions in Sr_2_Ca_0.88_Eu_0.02_Yb_0.04_Li_0.06_Mo_1–x_W_x_O_6_ (SCEYMWO for short) samples pumped by a 976 nm laser beam at various pumping powers and temperatures was conducted.^84^ The UC spectra are depicted in **Figure** **25**a and b,all of which are normalised to the ≈612 nm peak. The intensity ratios of the ^5^ *D*_0_→^7^ *F*_1_ magnetic dipole transition to the ^5^ *D*_0_→^7^ *F*_2_ electric dipole transition barely change for all samples, despite the different pumping powers and temperatures, which suggests the consistency of the coordination environment (site symmetry) of the Eu^3+^ sites in these samples owing to the sensitive Eu^3+^ ion probe. The ^5^ *D*_0_→^7^ *F*_4_ electric dipole transition shows apparent variation for samples with Mo^6+^, especially at different temperatures, which indicates a variety of polarisation in the environment around the Eu^3+^ ions rather than a change in the Eu^3+^ site symmetry. Because the electric field *E* of the 976 nm laser beam (≈10^14^ Hz) can only cause electronic polarisation when the SCEYMO and SCEYWO samples are stimulated by 976 nm laser light, theoretical calculations of the electronic band structure, density of states and optical properties were performed to determine contribution of electronic polarisation for these two samples. The extracted dielectric permittivities from the calculation results are 3.45 and 2.87 for SCMO and SCWO near the 0 eV point, respectively, as shown in Figure 25c and 25d, which suggests that SCMO is more easily polarised than SCWO. This result indicates that the tailoring behaviour of the ^5^ *D*_0_→^7^ *F*_4_ transition of Eu^3+^ is due to electronic polarisation of MoO_6_^6–^‐containing compounds upon laser beam stimulation. An analogous phenomenon is also observed in Eu^3+^‐doped zeolite‐Y for laser stimulation (to be published by our group). This research provides a novel technique to tailor the UC behaviour of Ln^3+^ ions through the electric field of a laser beam itself in addition to applying an external electric field, and varying the chemical composition and codopants, which might inspire the design of opto‐electric multifunctional devices.^84^

**Figure 26:**
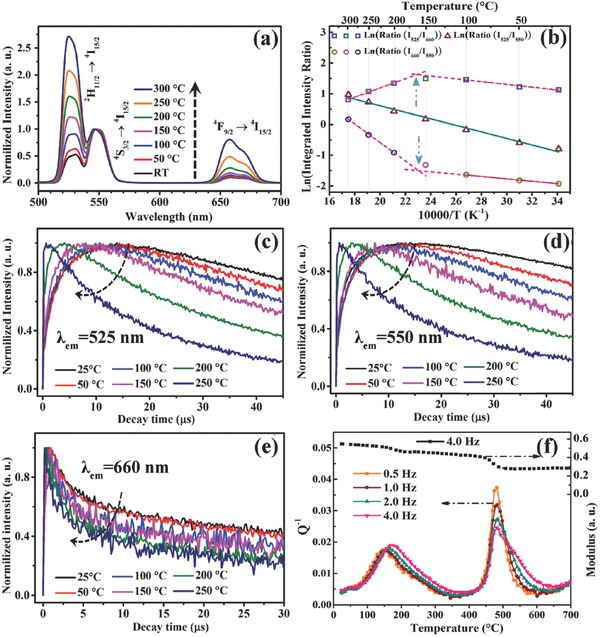
a) Temperature‐dependent UC emission spectra of La_2_Mo_2_O_9_:Yb^3+^,Er^3+^. All the spectra are normalized at ≈550 nm peak; b) the logarithm of peak intensity ratio versus 1/T; Temperature‐dependent UC luminescence decay curves of La_2_Mo_2_O_9_:Yb^3+^,Er^3+^ upon excitation of 980 nm pulsed laser monitored at c) λ_em_ = 525 nm, d) λ_em_ = 550 nm, e) λ_em_ = 660 nm; f) the internal friction and modulus versus temperature of the La_2_Mo_2_O_9_:Yb^3+^,Er^3+^ ceramics. Reproduced with permission.^87^ Copyright 2016, The Royal Society of Chemistry.

The coordination number variation of MoO_x_ groups in La_2_Mo_2_O_9_:Yb^3+^,Er^3+^, which results in oxide‐ion conductivity, also modulates the UC luminescence of Yb^3+^–Er^3+^.^87^ La_2_Mo_2_O_9_ is an outstanding oxide‐ion conductor, with a two orders of magnitude increase in conductivity at ≈580 °C, at which the monoclinic α‐La_2_Mo_2_O_9_ transforms to cubic β‐La_2_Mo_2_O_9_.^88^, ^89^ The oxide‐ion conductivity of β‐La_2_Mo_2_O_9_ originates from the structure of α‐La_2_Mo_2_O_9_. α‐La_2_Mo_2_O_9_ has a large unit cell with complex MoO_4_^2–^, MoO_5_^4–^, and MoO_6_^6–^ polyhedral forms, whereas β‐La_2_Mo_2_O_9_ has only MoO_6_^6–^ octahedra. Therefore, there is a tendency for MoO_4_^2–^ and MoO_5_^4–^ groups to transform to MoO_6_^6–^ groups with increasing temperature below the phase transformation point owing to the motion of the oxide ion,^89^ which is evidenced by the temperature‐dependent Raman spectra.^87^ The motion of the oxide ion influences UC luminescence, see **Figure** **26**. Yb^3+^–Er^3+^ ions are used because they are well‐studied, and the intensity ratio of the ^2^ *H*_11/2_→^4^ *I*_15/2_ (≈525 nm) to ^4^ *S*_3/2_→^4^ *I*_15/2_ (≈550 nm) transitions of the Er^3+^ ion is independent of the luminescence loss and fluctuations in the excitation intensity because of the small energy gap between ^2^ *H*_11/2_ and ^4^ *S*_3/2_. That is why the logarithm of the ratio (I_525_/I_550_) versus 1/T in Figure 26b is linear, which is the basis of optical temperature sensing applications. Both the logarithm of the ratio (I_525_/I_660_) and the ratio (I_660_/I_550_) versus 1/T have break points at 150–200 °C. The temperature‐dependent decay curves of the 525 and 550 nm UC emission of Er^3+^ behave distinctly above 150 °C compared with that of the 660 nm UC emission, as shown in Figure 26c–e. The *AC* impedance shows oxide‐ion long‐range motion above 200 °C but does not provide specific information below 200 °C owing to the limits of the instrument. Internal friction spectroscopy illustrates the short‐range oxide‐ion movement, as shown in Figure 26f. The peak near ≈150 °C is ascribed to the O^2–^ → V_o_ short‐range swapping and the peak at ≈500 °C is due to the phase transformation. Furthermore, the temperature‐dependent Raman spectra also show distinct behaviour below and above 200 °C. There is a correlation between the break points in Figure 26b–d and the friction peak at ≈150 °C in Figure 26f, which indicates that the oxide‐ion and oxygen‐vacancy swapping has a significant influence on the UC luminescence of Yb^3+^–Er^3+^ when the MoO_4_^2–^and MoO_5_^4–^ groups transform to MoO_6_^6–^ groups and that the UC luminescence could sense oxide‐ion motion. This system has potential in detecting the oxide‐ion motion of oxide‐ion conductors for applications in solid oxide fuel cells, oxygen sensors and oxygen separation.

## 4 Upconversion of *d* ^0^ ions

**Figure 27:**
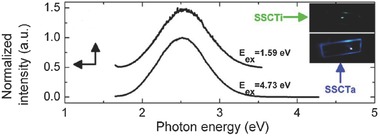
Emission spectra for Ti^4+^ doped silicate glass when excited by UV light at 4.73 eV and NIR femtosecond pulsed laser at 1.59 eV. The insets are images of Ti^4+^ doped silicate glass and Ta doped silicate glass irradiated by 780 nm femtosecond laser. Reproduced with permission.^93^ Copyright 2007, American Institute of Physics.

The Stokes luminescence of *d* ^0^ ion‐centred anion groups has been previously and intensively discussed by Blasse;^46^ however, the luminescence mechanism is not fully understood. In addition, the mechanism of the UC emission of *d* ^0^ ion‐centred anion groups remains unknown. The UC emissions of VO_4_^3–^, MoO_4_^2–^, WO_4_^2–^, TiO_x_^4–2x^ and TaO_x_^5–2x^ groups were observed in YVO_4_,^90^, ^91^ TiO_2_:Mo^92^ and silicate glasses with *d* ^0^ ion dopants^93^, ^94^, ^95^ when pumped by an infrared pulsed laser or continuous‐wave laser. One example of TiO_x_^4–2x^ emission is shown in **Figure** **27**. The origin of the emission from defects is excluded according to the experimental results.^93^ Therefore, there are two primary types of mechanisms for such a UC process. One mechanism involves low‐valent TM ions with an intermediate energy level, such as V^4+^/V^3+^ and Mo^4+^/Mo^5+^ in YVO_4_ 90 and TiO_2_:Mo,^92^ respectively, as a storage level for the subsequent UC process. The other mechanism involves multiphoton absorption induced by a femtosecond pulsed laser in *d* ^0^‐ion‐doped glass or crystal,^91^, ^93^, ^94^, ^96^ resulting in electron excitation from the 2*p* orbitals of O^2–^ to the *d* orbitals of *d* ^0^ TM ions and UC emission with the reverse process. This result is deduced from the fact that there is no intermediate energy level available in the absorption spectra, which is requisite for a UC process through an ESA or ETU mechanism. Additionally, the power of the femtosecond pulsed laser is sufficiently high and the pulse is sufficiently fast to generate a nonlinear effect with multiphoton absorption. This type of material may find application in high‐density optical storage and three‐dimensional colour displays.

## 5 Conclusions and Perspectives

In contrast to the well‐known multiple and fixed UC emissions of Ln^3+^ in Ln^3+^‐activated luminescent materials, TM ions with non‐filled *d* orbitals feature single and tuneable broadband UC emission owing to the susceptibility of the *d* electron energy levels of TM ions to their chemical environment. The UC emissions of Ln^3+^ can also be modulated by TM and *d* ^0^ ions (anion groups), which is appealing because it benefits from the specific metastable energy levels of Ln^3+^, independent of the ligand field and the tuneable energy levels of TM ions or the electric versatility of *d* ^0^ ion‐contained hosts. Significant advances have recently been made in fabricating new luminescent materials, developing novel applications and exploring the mechanisms involved.

(1) For most TM ions, their UC emissions are normally quenched at RT owing to the environment‐sensitive abundant *d* energy levels and strong phonon‐electron coupling. Some exceptions, including Mn^2+^ and Cr^3+^, are ascribed to the large gap between the first excited state and the ground state (>10,000 cm^–1^). The large gap decreases the possibility of multiphonon relaxation. Meanwhile, a large gap without metastable energy level that matches the excited state ^2^ *F*_5/2_ of Yb^3+^ at ≈10,000 cm^–1^ for resonant energy transfer requires cooperative sensitisation or GSA/ESA(ETU) based on a superexchanged coupling model for Yb^3+^–TM ions. The most appealing feature of Mn^2+^/Cr^3+^ is the tuneable single‐band emission, which is attractive for bioimaging. Furthermore, the susceptibility of the *d* electrons of TM ions may make them magnetically coupled with each other or Ln^3+^, resulting in new physical processes with multifunctionality, as in the case of Mn^2+^. Since the outstanding magnetism property and excellent luminescence performance are normally contrary and always conflicts, the options of TM ions are limited. For those TM ions with abundant energy levels and small gap between the adjacent levels, the excited TM ions stimulated by NIR light via upconverted process would not result in UC emission but might cause photocatalysis when they locate at proper sites at the surface of the materials like nanocrystals for catalysis application.
(2) For the UC emissions of Ln^3+^ modulated by TM ions, the options of TM ions are limited to Mn^2+^ and Cr^3+^ because of the large gap mentioned above and the lack of energy levels below 10,000 cm^–1^ to quench the UC of Ln^3+^. They generally enhance the emission of Ln^3+^ that with emission levels below the emitting states of Mn^2+^/Cr^3+^ owing to energy transfer. The research provides an effective approach to modulate the multiple emissions of Ln^3+^ to single‐band emission, which greatly increases the signal‐to‐noise ratio when applied in bioimaging. Also, the introduction of TM ions would facilitate the applications of these systems in the magnetism‐related field due to the magnetic coupling of TM–TM ions or TM–Ln^3+^ ions. It is highly desirable to utilise the broadband absorption characteristics of TM ions to sensitise Ln^3+^ UC emissions for applications in solar cells because it would increase the absorption cross section. However, much effort on controlling the spatial distribution of TM and Ln^3+^ ions to a certain distance, such as constructing a core‐shell structure, is required to prevent mutual quenching to further improve the luminescence efficiency.
(3) For the UC emissions of Ln^3+^ modulated by *d* ^0^ ions (anion groups), the electric versatility of *d* ^0^‐contained hosts has a strong but unspecific influence on some electric dipole transitions of Ln^3+^. This is mostly ascribed to the unknown details of the *d* ^0^‐centred anion groups that coordinate Ln^3+^. There is little research on the UC luminescence of the *d* ^0^‐centred anion groups themselves. However, functions in addition to the UC property are indeed introduced in these systems, such as ferroelectricity and oxide‐ion conductivity. We could imagine further to couple the UC emission of Ln^3+^ ions in piezoelectric compounds, negative thermal expansion compounds and polar compounds to enlarge the UC luminescent material family and promote multifunctional applications.

Overall, the attractive electronic and magnetic behaviour of TM ions and the electric behaviour of *d* ^0^‐contained hosts make them beneficial in applications involving multifunctional materials and devices when combined with the UC behaviour of Ln^3+^. There remains room for more imaginative and innovative research work, which allows researchers to be creative with the mechanism and application of these materials.
